# The auxin receptor TIR1 homolog (PagFBL 1) regulates adventitious rooting through interactions with Aux/IAA28 in *Populus*

**DOI:** 10.1111/pbi.12980

**Published:** 2018-07-24

**Authors:** Wenbo Shu, Houjun Zhou, Cheng Jiang, Shutang Zhao, Liuqiang Wang, Quanzi Li, Zhangqi Yang, Andrew Groover, Meng‐Zhu Lu

**Affiliations:** ^1^ State Key Laboratory of Tree Genetics and Breeding Research Institute of Forestry Chinese Academy of Forestry Beijing China; ^2^ Guangxi Academy of Forestry Nanning Guangxi China; ^3^ US Forest Service Pacific Southwest Research Station Davis CA USA

**Keywords:** adventitious root development, auxin signalling, *PagFBL1*, *PagIAA28*

## Abstract

Adventitious roots occur naturally in many species and can also be induced from explants of some tree species including *Populus*, providing an important means of clonal propagation. Auxin has been identified as playing a crucial role in adventitious root formation, but the associated molecular regulatory mechanisms need to be elucidated. In this study, we examined the role of *PagFBL1*, the hybrid poplar (*Populus alba* × *P*. *glandulosa* clone 84K) homolog of *Arabidopsis* auxin receptor TIR1, in adventitious root formation in poplar. Similar to the distribution pattern of auxin during initiation of adventitious roots, *PagFBL1* expression was concentrated in the cambium and secondary phloem in stems during adventitious root induction and initiation phases, but decreased in emerging adventitious root primordia. Overexpressing *PagFBL1* stimulated adventitious root formation and increased root biomass, while knock‐down of *PagFBL1* transcript levels delayed adventitious root formation and decreased root biomass. Transcriptome analyses of *PagFBL1* overexpressing lines indicated that an extensive remodelling of gene expression was stimulated by auxin signalling pathway during early adventitious root formation. In addition, *PagIAA28* was identified as downstream targets of PagFBL1. We propose that the *PagFBL1*‐*PagIAA28* module promotes adventitious rooting and could be targeted to improve *Populus* propagation by cuttings.

## Introduction

Roots play a crucial role in water and nutrient acquisition to support growth of the aerial parts of the plant, and healthy root systems contribute in maximizing plant biomass (Jansen *et al*., 2013). In contrast to lateral roots (LRs) that occur on primary roots and originate from pericycle cells of primary roots, adventitious roots (ARs) can be formed from above‐ground organs such as leaves, hypocotyls and stems, and are initiated from cambial or adjacent vascular cells (Legué *et al*., 2014; Verstraeten *et al*., 2014). AR founder cells are believed to dedifferentiate from nonroot differentiated tissues (Srivastava, 2002). AR formation occurs naturally in most monocotyledonous species and many species of tropical and temperate wet forest trees as a part of the normal development. Commercially, ARs are produced during vegetative propagation by artificial induction using wounding or hormone application treatments in many dicotyledonous species (Nadkarni, 1994; Pacurar *et al*., 2014). The biological processes involved in AR formation are complex, and the temporal phases can be described as induction, initiation, activation of root primordium and out‐growth (Legué *et al*., 2014). These processes are influenced by multiple factors, such as the genetic background and the physiological status of the mother plants, the application of hormones and environmental conditions (Geiss *et al*., 2010; Pacurar *et al*., 2014).

Phytohormones are the most important modulators of AR development (Bellini *et al*., 2014). Plant hormones, such as abscisic acid (Da Costa *et al*., 2013; Mehrotra *et al*., 2014), cytokinin (Della Rovere *et al*., 2013), ethylene (Muday *et al*., 2012; Negi *et al*., 2010), gibberellin (Mauriat *et al*., 2014; Niu *et al*., 2013), jasmonic acid (Da Costa *et al*., 2013) and strigolactones (Rasmussen *et al*., 2012; Sun *et al*., 2015), form a signalling network influencing cell fate determination and specification in which auxin plays the crucial role (Da Costa *et al*., 2013; Pacurar *et al*., 2014; Pop *et al*., 2011). Early in the 1930s, indole‐3‐acetic acid (IAA) was shown to be effective in promoting the formation of AR primordia (Thimann and Koepfli, 1935), and since then IAA has been widely used to induce AR formation in the clonal propagation of various tree species, including poplar (Preece, 2003; Rademacher *et al*., 2011). On the other hand, anti‐auxin agents applied at AR early phases causes significant inhibition of AR in poplar cuttings (Bellamine *et al*., 1998). In addition, the IAA content of an easily rooted genotype was higher than of a difficult to root genotype of *Eucalyptus globulus* (De Almeida *et al*., 2015; Negishi *et al*., 2011). These observations have demonstrated the important role of auxin in AR induction.

It is well‐established that auxin is perceived by a receptor (SCF^TIR1/AFB^), which upon binding auxin targets AUXIN/INDOLE‐3‐ACETIC ACID INDUCIBLE (Aux/IAA) proteins for degradation. Aux/IAA proteins repress auxin response factors (ARFs), the latter activate or repress downstream auxin signalling genes upon released from repression Aux/IAAs (Dharmasiri *et al*., 2005; Kepinski and Leyser, 2005; Chapman and Estelle, 2009; Wang and Estelle, 2014; Korasick *et al*., 2015; Salehin *et al*., 2015). The three key signalling elements TIR1/AFBs, Aux/IAAs and ARFs are encoded by gene families of 6, 29 and 23 members in *Arabidopsis* (Chapman and Estelle, 2009) and 8, 35 and 39 in *Populus* (Brunner *et al*., 2007), respectively. A different context of *ARF* and *Aux/IAA* gene expression can lead to a differential auxin signalling (Quint and Gray, 2006); thus, it is important to find out the partners of TIR1, Aux/IAA and ARF members, which execute a given auxin signalling pathway. The induction of auxin‐inducible acyl amido synthetases, Gretchen Hagen 3 (GH3), by the ARF family (Zhang *et al*., 2016) is the early event of such a signalling cascade.

In the past few decades, significant progress has been made in the regulation of root development by auxin in *Arabidopsis* (Petricka *et al*., 2012; Ubeda‐Tomás *et al*., 2012). AR formation, in contrast, has proved difficult to study, and the mechanisms controlling AR initiation and development are poorly understood. Recent studies in *Arabidopsis* showed that auxin is likely to induce AR initiation through the activation of an auxin signalling network similar to that in LR (See the recent review by Bellini *et al*., 2014). In LR formation, there are two signalling pathways, namely TIR1/AFB2‐IAA12,28‐ARF5 and TIR1‐IAA14‐ARF7,19 (Bellini *et al*., 2014). ARF6, 8 and 17 have been identified in auxin signalling pathways during AR formation in *Arabidopsis*, but which SCF^TIR1/AFB^ or Aux/IAA members are involved in this signalling process remains elusive (Bellini *et al*., 2014; Gutierrez *et al*., 2012). Characterization of rice and maize mutants that are altered in AR and LR development showed that the transcriptional regulatory pathway is conserved in cereals and *Arabidopsis*, involving TIR1/AFB2 auxin receptors and the Aux/IAA, ARF and LBD (LATERAL ORGAN BOUNDARIES DOMAIN) transcription factors (Ormanligeza *et al*., 2013). Analysis of gene expression in poplar cuttings indicated that the context of genes encoding Aux/IAA and ARF proteins were remodelled during the first 2 days after excision of stems (Ramírez‐Carvajal *et al*., 2009), and some ARF family members were specifically expressed during adventitious rooting in *P. trichocarpa*, based on transcriptomic data (Rigal *et al*., 2012).

Understanding AR formation in trees is important (Legué *et al*., 2014), because this capability underlies the ability to vegetatively propagate millions of cuttings from elite clones for commercial production (Li *et al*., 2009). Recent advances in *Populus* suggest that it is feasible to identify genes and their pathway regulating adventitious rooting, which are remodelled in cells prior to AR initiation (Ramirez‐Carvajal and Davis, 2010; Verstraeten *et al*., 2013). It is anticipated that the underlying mechanisms of some developmental aspects of the induction and formation of ARs may be common between *Arabidopsis* and *Populus* (Verstraeten *et al*., 2013). However, whether it will be possible to translate what is known about AR development in herbaceous species to woody species still needs to be investigated (Bellini *et al*., 2014).

We previously performed a comprehensive analysis of the poplar auxin receptors and found the *TIR1* homolog *PtrFBL1* from *P*. *tricocarpaa* plays an important role in growth rate and development (Shu *et al*., 2015). Here, we report that FBL1 (PagFBL1) from hybrid poplar (*P*. *alba* × *P*. *glandulosa*) clone 84K regulates AR formation from stem cuttings. We demonstrate that PagFBL1 is a key regulator in auxin signalling pathway to induce adventitious rooting, and the potential downstream regulators, including candidate Aux/IAAs in the auxin signalling pathway, are also identified in poplar.

## Results

### *PagFBL1* exhibits spatially distinct expression patterns during adventitious rooting

**Figure 1:**
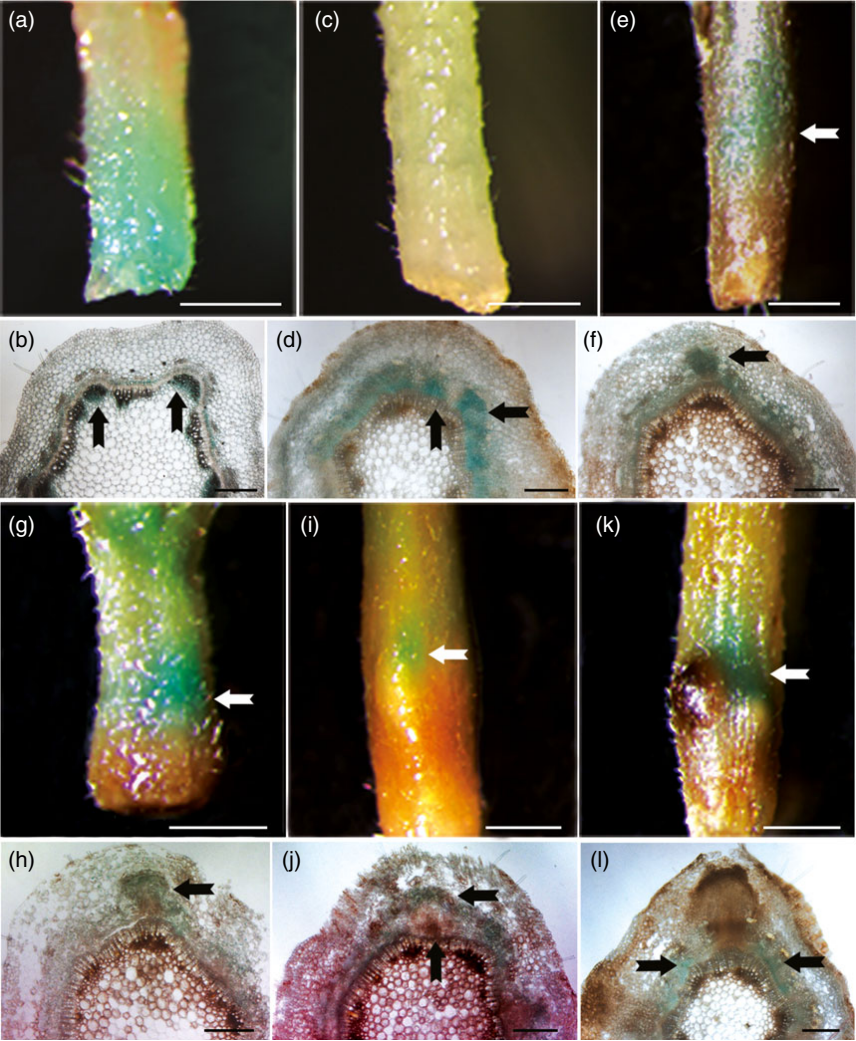
Expression patterns of *PagFBL1* during AR formation. GUS staining of Pro_*Pag*_ _*FBL*_ _*1*_::GUS leafy stems (a, c, e, g, i, k) and their transverse sections (b, d, f, h, j, l); the samples were collected at 0 day (a, b), 2 days (c, d), 3 days (e, f), 4 days (g, h) 5 days (i, j) and 6 days (h, l). Experiments were repeated three times for each, and the representative phenotypes are shown. Scale bars: (a, c, e, g, i, k) 1 mm; (b, d, f, h, j, l) 200 μm.

The expression patterns of genes can provide useful clues to their functions. Therefore, we generated *Ppag*::*GUS* transgenic 84K plants to investigate *PagFBL1* expression pattern (Materials and methods). GUS signal was mainly observed in the cambial zone and immature xylem in stems at time zero after cutting (Figure 1a, b), and then became more broadly expressed in the cambium zone and secondary phloem 2 days after AR induction (Figure 1c, d). Three to four days after AR induction, GUS signal was observed in the AR primordium which included cells within the cambial zone, secondary phloem and cortex (Figure 1e–h). GUS signal decreased within the enlarged root primordium by 5 days after AR induction (Figure 1i, j) and was undetectable within the AR 6 days after AR induction (Figure 1k, l). This indicates that *PagFBL1* may be involved in the formation of ARs at early stages, *that is* induction and initiation phases. In addition, in comparison with our earlier study on AR formation using DR5::GUS auxin response reporter lines, the expression of *PagFBL1* showed similar dynamic changes with the auxin distribution during AR formation (Liu *et al*., 2014). These results suggest that the PagFBL1, the auxin receptor, could participate in the auxin signalling pathway to regulate AR induction and initiation.

### Overexpression and knock‐down of *PagFBL1* affect AR formation in transgenic poplar

**Figure 2:**
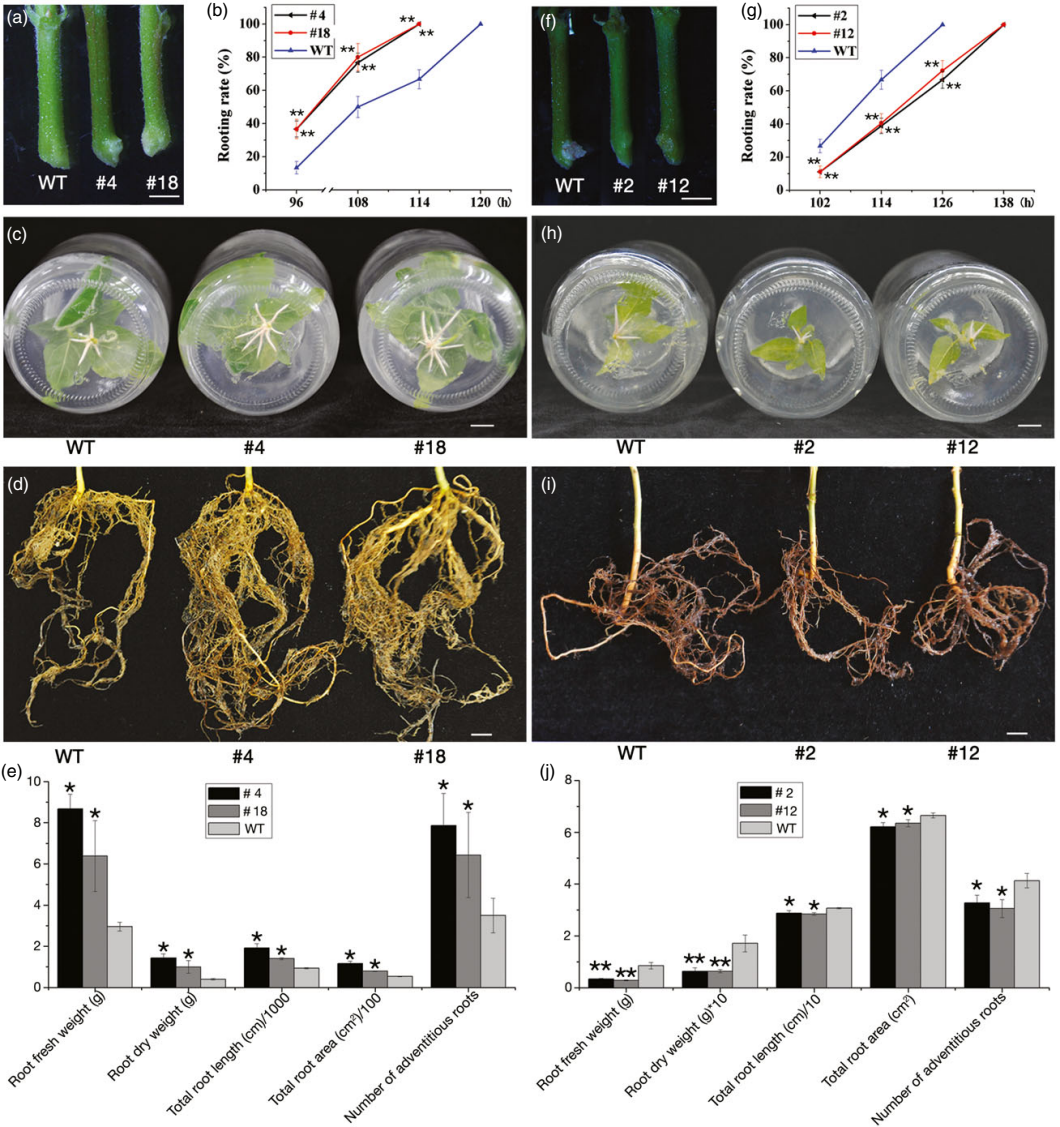
ARs from leafy stems of *PagFBL1* overexpressed lines #4 and #18, knock‐down lines #2 and #12 and WT. (a–e) for #4 and #18: (a) the early stage of ARs. (b) Rooting rates as the percentage of leaf stem explants with emerged ARs. (c) Number of AR induced. (d) AR system from 5 months plants in soils. (e) The quantification of ARs from 5 months plants; (f**–**g) for #2 and #12: (f) the early stage of ARs. (g) Rooting rates. (h) Number of AR induced. (i) AR system from 2 months plants in soils. (j) The quantification of ARs from 2 months plants. Bars = 1 cm. The values are means ± SE of three replicates. Significant differences between WT and transgenic lines are indicated with asterisks (*P <* *0.05 and **P <* *0.01).

The expression of *PagFBL1* during early AR development prompted us to study its role in this process. In total, 19 independently transformed overexpression (OE) lines and 20 lines with knock‐down transcript levels (KD) lines were generated, and the relative up‐ and downexpression of *PagFBL1* in these lines was quantified using real‐time qRT‐PCR (Materials and methods). We selected eight OE lines and eight KD lines with moderate change in expression levels, respectively (Figure S1a), and investigated their rooting ability. The OE lines exhibited earlier AR emergence (Figure 2a, Figure S1b), higher percentage of leafy stem explants with ARs at different times after induction and 6 h earlier in reaching to 100% than wild‐type controls (WTs) (Figure 2b, Figure S1c). In addition, the number of ARs generated from leafy stem explants of OE lines was significantly increased (Figure 2c, e) and supported larger root systems (Figure 2d) as measured by total root length, root area, fresh and dry weight (Figure 2e) 5 months after planting in soil. OE lines produced earlier and more ARs than WTs, and this phenomenon was even pronounced under IAA treatment using leaf explants (Figure S2a‐e). In *PagFBL1* KD lines, the emergence time was delayed (Figure 2f, g, Figure S1d, e), even under IAA treatment using leaf explants (Figure S2f‐h). In addition, the number (Figure 2h, j) and biomass of ARs (Figure 2i, j) were significantly decreased compared with WTs as well as OE lines. These findings suggest that *PagFBL1* plays a significant role in AR formation in poplar.

### Overexpressing *PagFBL1* stimulates the remodelling of gene expression in transgenic poplar

**Figure 3:**
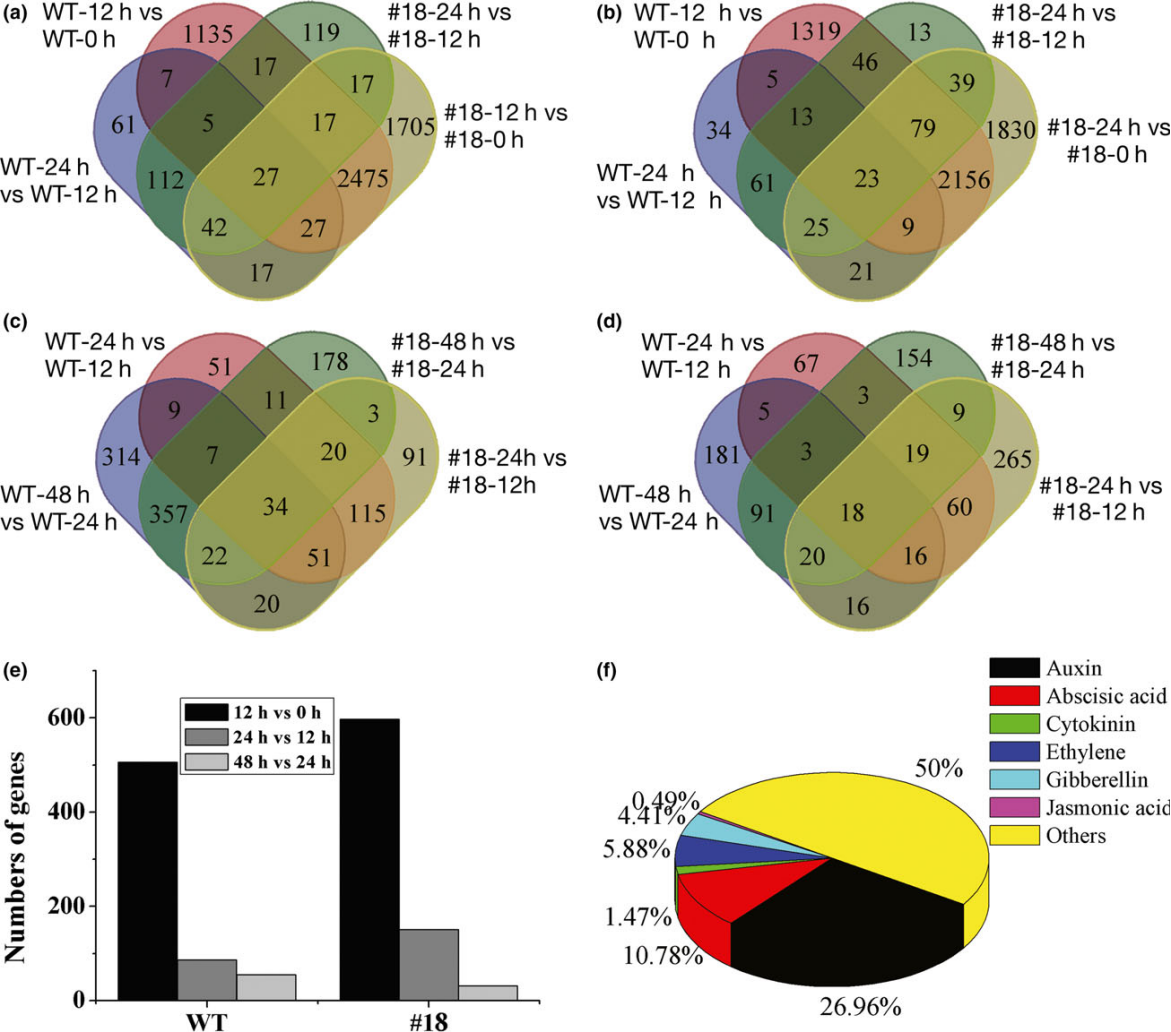
Venn diagrams showing the number of DEGs classified into groups of 0, 12, 24, 48 h after AR induction. (a) Up‐regulated genes from 12 h vs 0 h and 24 h vs 12 h. (b) Down‐regulated genes from 12 h vs 0 h and 24 h vs 12 h. (c) Up‐regulated genes from 24 h vs 12 h and 48 h vs 24 h. (d) Down‐regulated genes from 24 h vs 12 h and 48 h vs 24 h. (e) COG classification of DEGs in signal transduction mechanisms. (f) DEG percentages for major hormones in plant hormone signal transduction based on KEGG pathway.

To gain molecular insights into the roles of *PagFBL1* in adventitious rooting, a transcriptome analysis was performed using RNA sequencing to identify differentially expressed genes (DEGs) in AR formation (Materials and methods). For WT, a total of 8855 genes were significantly differently expressed between 0 and 12 h after AR induction, with 4488 up‐regulated and 4367 down‐regulated in nontransgenic controls (Figure 3a, b). However, only 1,607 DEGs including 881 up‐ and 726 down‐regulated genes were detected from 12 to 24 h after induction (Figure 3a, b), and 1121 DEGs with 814 up‐ and 350 down‐regulated from 24 to 48 h were obtained (Figure 3c, d). Similarly, for OE line #18, a total of 10 373 DEGs with 5357 (2546 shared with WT) up‐ and 5,016 (2267 shared with WT) down‐regulated were detected from 0 to 12 h (Figure 3a, b). Only 2441 DEGs including 1099 up‐ and 1342 down‐regulated (Figure 3a, b) from 12 to 24 h, and 949 DEGs with 632 up‐ and 317 down‐regulated from 24 to 48 h were found after AR induction (Figure 3c, d). The numbers of DEGs between 0 and 12 h in both non‐ and transgenic plants were much larger than that between 12 and 24 h or 24 and 48 h. Therefore, the remodelling of expression of a larger numbers of genes occurred in the first 12 hours of AR induction and initiation. Notably, 1518 more DEGs appeared in first 12 h in the OE #18 line, 119 of which appeared later (from 12 to 24 h) in WT (Figure 3a, Table S1). These results suggest that the high level of PagFBL1 could potentiate the shift of gene expression in favour of AR formation.

**Figure 4:**
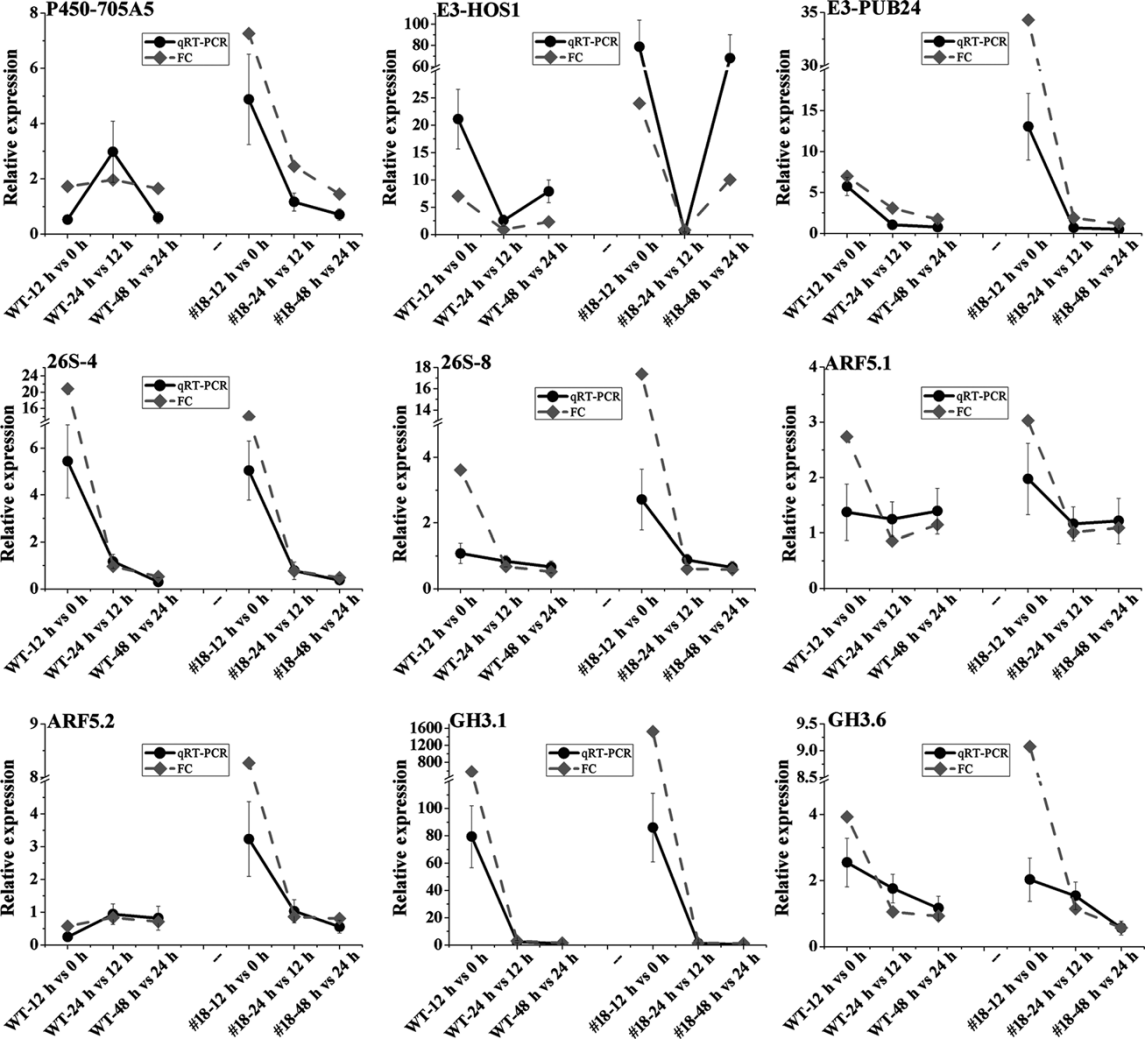
Expression profiles of the genes (Table S2) related to auxin signalling pathways at different time points during AR formation by both qRT‐PCR and RNA‐Seq (fold change for FPKM).

To understand the significance of DEGs, clusters of orthologous groups (COG) classification was determined (Figure S3a‐f). A large number of genes involved in signal transduction mechanisms were induced in the first 12 h, comparing to the other time points both in OE line #18 and WT (Figure 3e, Figure S3). We also mapped the DEGs to the KEGG pathway database to investigate their functions and found that a large number of DEGs were only enriched in plant hormone signal transduction in the first 12 h in #18 (Figure S4d). Interestingly, the largest proportion of DEGs (26.9%) were involved in the auxin signalling pathway, compared to other hormones (Figure 3f). These genes included five cytochrome P450 members, one Cullin‐1 (CUL1), seven E3 ubiquitin‐protein ligases, four 26S PROTEASOME proteins, two IAA28 members, three ARFs and three GH3 members (Table S2). The expression of these genes (in WT and #18) using qRT‐PCR also showed similar trends as obtained by the RPKM (reads per kilobase of exon model per million mapped reads) based on RNA sequencing (Figure 4), but *ARF5.1* showed notable difference in the first 12 h after induction. As many more samples were collected for RNA sequencing than for qRT‐PCR, the expression data based on RNA sequencing were more reliable. *ARF5.1*,* ARF5.2*,* GH3.1* and *GH3.6* exhibited high expression in the AR induction phase based on the RNA sequencing data both in OE and KD lines, and this was more pronounced in OE lines. The involvement of IAAs and ARFs during AR induction suggests that auxin promotes AR formation at the induction and initiation phases through FBL1‐IAA‐ARF signalling.

### *PagFBL1* executes auxin signalling by interacting with PagIAA28.1 and PagIAA28.2

Auxin signalling starts from the FBL1‐mediated Aux/IAA degradation, prompting us to identify Aux/IAA proteins that might be targeted by PagFBL1. *Aux/IAA* genes exhibit differential expression under auxin treatment and are generally more responsive than *ARF* or *TIR1/AFB* genes (De Almeida *et al*., 2015; Ivan *et al*., 2008; Trenner *et al*., 2016; Villacorta‐Martín *et al*., 2015; Wen *et al*., 2016; Xu *et al*., 2017a). We screened the candidate *Aux/IAA* genes that showed changes in expression during the early stages of AR formation, or else show differential expression in WT control vs OE plants undergoing AR formation. Based on the transcriptome in this study (Figure S5) and that in *P. trichocarpa* (Ramírez‐Carvajal *et al*., 2009), we selected 15 genes (and their alternative transcripts) with such expression patterns during AR formation, including *PagIAA7.1*,* PagIAA7.2*,* PagIAA9*,* PagIAA12.1*,* PagIAA16.1*,* PagIAA16.2*,* PagIAA16.3*,* PagIAA16.4*,* PagIAA19.1*,* PagIAA20.1*,* PagIAA27.1*,* PagIAA28.1*,* PagIAA28.2*,* PagIAA29.2* and *PagIAA29.3*. Their expression was further checked during AR formation in both WT and #18 using qRT‐PCR (Figure S6). The results showed all these genes’ transcript levels were significantly changed in during AR induction in WT and #18 (Figure S6); thus, their proteins were then tested as candidate targets of PagFBL1.

**Figure 5:**
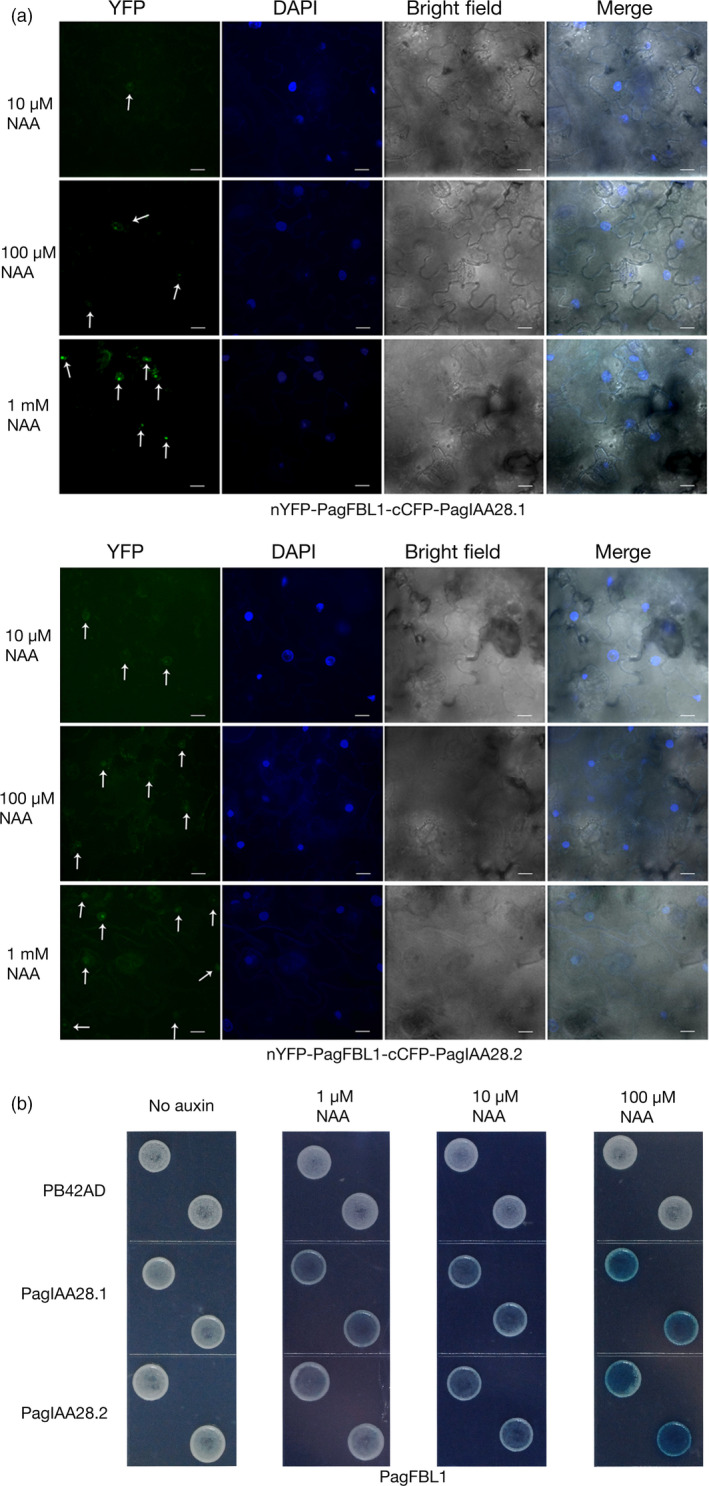
Interactions of PagFBL1 and PagIAAs revealed by BiFC assay and LexA yeast two‐hybrid assay. (a) PagFBL1 and PagIAA 28.1 or 28.2 by BiFC assay. Bars = 12 μm. (b) PagFBL1 and PagIAA28.1 or 28.2 by LexA yeast two‐hybrid assay.

To determine which PagIAA members are targeted by PagFBL1, we used a bimolecular fluorescence system, in which PagFBL1 and one of the 15 PagIAA members were fused to each half of the yellow fluorescence protein (YFP) and co‐expressed transiently in tobacco leaves (Materials and methods). The complemented YFP fluorescence signals were checked for the 15 combinations tested (Figure 5a, Figure S7). The YFP signal was only observed in the nucleus (merged with DAPI signal) when nYFP‐PagFBL1 was cotransformed with cCFP‐PagIAA28.1 or cCFP‐PagIAA.28.2 (Figure 5a), and intensified in higher auxin concentration, but only DAPI signals were observed in other combinations (Figure S7).

Previous studies showed that the LexA yeast two‐hybrid system can be used to study the interaction between auxin receptors TIR1/AFB and their substrates Aux/IAAs (Calderón Villalobos *et al*., 2012; Yu *et al*., 2015). To further verify the above interactions identified by the bimolecular fluorescence system, PagFBL1 was fused to the LexA DNA‐binding domain and introduced into a strain expressing the PagIAA28.1 or PagIAA28.2 protein fused with LexA activating domain. The interaction between PagFBL1 and PagIAA28.1 or PagIAA28.2 was confirmed and its strength increased as measured by galactosidase activity following the elevated auxin concentrations (Figure 5b). This result demonstrates that PagFBL1 can interact strongly with both PagIAA28.1 and PagIAA28.2 in the presence of NAA, thus are candidates for participating in AR induction in poplar stem segments.

## Discussion

The mechanisms underlying AR formation and the cause for variation among plant species and genotypes in AR production are poorly understood (Hu and Xu, 2016; Liu *et al*., 2014; Sena *et al*., 2008). The details of the mechanisms underlying AR formation are of interest by virtue of their relevance to basic plant biology, but are also crucial for applied aspects for commercial woody plants, like poplar, for propagation of superior cultivars and capture of both additive and nonadditive genetic variance in tree improvement programmes (Dickmann *et al*., 2006). In this study, we provided details about the roles of auxin signalling pathways involved in regulation of AR formation in poplar.

In this study, we found the presumed poplar auxin receptor‐encoding gene, *PagFBL1*, was expressed in the cambium zone and secondary phloem during the AR induction phase to the early AR primordium formation, but down‐regulated in the enlarged AR primordium. This is a similar pattern to auxin distribution during AR induction (Liu *et al*., 2014). Previous studies suggest that strong auxin signalling is required during the induction phase of both LR and AR in *Arabidopsis* (Bustilloavendaño *et al*., 2017; De Klerk and De Jong, 1999; Du and Scheres, 2017; López‐Bucio *et al*., 2015; Sánchez *et al*., 2007). In woody plants, IAA was mostly located in the cambial region of rooting‐competent pine hypocotyls, and IAA content was higher in the cambium zone in *E. grandis* during the initial 24 h of AR induction (Abarca *et al*., 2014; De Almeida *et al*., 2015). In addition, compared to the hard‐to‐root species *E. globulus*, the easy‐to‐root species *E. grandis* had a peak of *TIR1* expression after 6 h of exposure to exogenous auxin (De Almeida *et al*., 2015), and *FB1* (homolog of *TIR1*) was also involved in the early induction of AR primordium by auxin and regulated the elongation of ARs by auxin in *Liriodendron* hybrids (Zhong *et al*., 2016). Together these results show that the auxin receptor FBL1 follows auxin distribution and acts at the very beginning of adventitious rooting. Indeed, overexpression of *PagFBL1* dramatically stimulated early AR formation and led to high number of ARs in OE poplars (Figure 2). Therefore, we conclude that PagFBL1 may serve as a key regulator promoting the formation of root primordia in poplar.

The initiation stage of AR is characterized by cell division and organization of the root primordia (Li *et al*., 2009). Studies in apple (De Klerk and De Jong, 1999; De Klerk *et al*., 1995), chestnut (Sánchez *et al*., 2007), *Populus* (Ribeiro *et al*., 2016; Rigal *et al*., 2012), *Petunia* (Ahkami *et al*., 2014; Druege *et al*., 2014), carnation (*Dianthus caryophyllus*) (Villacorta‐Martín *et al*., 2015), *Catalpa bungei* (Wang *et al*., 2016) and mung bean (Steffens and Rasmussen, 2016) reveal that the critical events that culminate in the formation of ARs in cuttings occur in the first 3–24 h, and the induction stage comprises molecular and biochemical events without visible changes. Auxin signal transduction was revealed in the transcriptome during the formation of ARs in cuttings in the first 24 h after induction of *Populus* (Ramírez‐Carvajal *et al*., 2009), *Petunia* (Ahkami *et al*., 2013; Druege *et al*., 2014), mung bean (*Vigna radiata*) (Li *et al*., 2015), carnation (Villacorta‐Martín *et al*., 2015) and *Malus xiaojinensis* (Xu *et al*., 2017b). These transcriptome analyses in AR induction stage provide a meaningful tool for investigating the auxin signalling pathway to regulate AR formation. Higher concentration of IAA is required in the AR induction stage to stimulate auxin‐induced cell division but may not be required during root meristem organization (Goldfarb *et al*., 2010); thus, auxin signal transduction was strengthened in the initiation of ARs of cuttings. In this study, consistent with the above observations, significant changes in gene expression patterns were found, particularly in the induction phase. DEGs enriched in plant hormone signal transduction were predominant at this stage. This also illustrates that a fast reprogramming of gene expression is required to support AR formation, with plant hormone signalling playing a critical role. In addition, OE lines exhibited more DEGs in auxin signalling pathways in the early induction stage, which appeared later in WT plants (Figure 3f, Figure S4). This result is consistent with the earlier formation and higher number of ARs in OE lines. These results further emphasize that *PagFBL1* can stimulate this transition by strengthening the auxin signalling pathway.

It is well documented that auxin is perceived by SCF^TIR1/AFB^‐Aux/IAA complexes, and releases bound ARFs to regulate auxin‐mediated gene transcription through the degradation of Aux/IAA repressors (Dharmasiri *et al*., 2005; Kepinski and Leyser, 2005; Salehin *et al*., 2015). Although Aux/IAA family proteins are highly redundant, they have different affinity to TIR1 and drive degradation at varied rates, leading to different responses to auxin in diverse biological processes (Chen *et al*., 2017). Therefore, we further investigated which IAAs are targeted by PagFBL1 to activate auxin signalling. We found that only PagIAA28.1 and PagIAA28.2 interacted with PagFBL1 using BiFC assay and LexA yeast two‐hybrid assay in the presence of IAA in a dose‐dependent fashion. In *Arabidopsis*,* iaa28*‐1 mutant showed reduced AR formation (Bustilloavendaño *et al*., 2017; López‐Bucio *et al*., 2015), suggesting IAA28 is required to be degraded in order to release ARFs to initiate both LR and AR formation. Supporting to this suggestion, we found that it is necessary to degrade IAA28 to initiate auxin signalling in AR formation in poplar. Previous studies have also shown that unlike most known auxin‐inducible Aux/IAAs, both IAA28 expression levels and protein abundance have been reported to be reduced by auxin treatment in the LR formation (De Rybel *et al*., 2010; Parizot *et al*., 2010; Rogg *et al*., 2001). In addition, IAA28 was suggested to release ARF5, ARF6, ARF7, ARF8 and ARF19 in LR initiation in *Arabidopsis* (De Rybel *et al*., 2010), and three of them (PagARF5.1, PagARF5.2 and PagARF7.3) were found specifically expressed in AR initiation in poplar in this study, indicating high level of these ARFs is required in AR initiation. Except the release of IAA‐bond ARFs mediated by FBL1, high expression of these ARFs may also be needed in the initiation of ARs. Indeed, *PagARF5.1* and *PagARF5.2* were highly expressed in OE lines comparing to WT lines, which may contribute to their early AR initiation and higher number of ARs, although the positive regulation of these ARFs in FBL1 OE lines needs to be elucidated. The induction of auxin‐inducible GH3 by the ARF family (Zhang *et al*., 2016) is an early event in the auxin signalling cascade. Previous studies have found that auxin‐inducible Gretchen Hagen3 (GH3) genes, GH3.3, GH3.5 and GH3.6, are required for fine‐tuning the AR initiation by modulating JA homoeostasis and regulated by ARF6, ARF8 and ARF17 in *Arabidopsis* (Gutierrez *et al*., 2012; Sorin *et al*., 2006). In this study, PagGH3.1, PagGH3.5 and PagGH3.6 were found up‐regulated during AR initiation in poplar and even pronounced in OE lines based on the RNA sequencing (Table S2, Figure 4), suggesting GH3 plays a key role in auxin signalling in both *Arabidopsis* and poplar. Due to the biological similarity between the initiation process of LRs and ARs (Legué *et al*., 2014; Verstraeten *et al*., 2014), the same signalling module may be shared in both processes. Our results thus provide an evidence that the FBL1‐IAA28.1,2‐dependent auxin signalling module involves in regulation of AR induction in poplar, which shares at least partly with mechanism in LR formation in *Arabidopsis*.

This study addresses the role of auxin in AR formation in poplar and suggests that FBL1 participates in an FBL1‐IAA28.1,2 module regulating AR formation in poplar, which shares similarity with the regulatory mechanisms of LR induction in *Arabidopsis*. PagFBL1 acts in auxin signalling required early in AR development, representing a potential biotechnological target for the improvement of poplar propagation by cuttings.

## Materials and methods

### Plant materials and growth conditions

*P*. *alba* × *P*. *glandulosa* clone 84K was used as the plant material for the cloning of *PagFBL1* and its transformation. Plants were propagated by microcuttings in bottles and cultured on 1/2 ×  MS (Murashige and Skoog) medium at 24 ± 1 °C under cool‐white light (60 ± 5 μmol photons m^2^/s at the top leaves surface, 16‐h light/8‐h dark) (Shu *et al*., 2015), and leafy stems from 3‐week‐old plants were used in induction experiments for ARs.

To reveal the role of *PagFBL1* in AR formation, we used a *PagFBL1* promoter::GUS assay to monitor the expression of *PagFBL1* during AR formation in a time course after AR induction from leafy stem segments. A 2.0 kb 5′‐UTR fragment of *PagFBL1* (KY020444) was amplified from the genomic DNA of 84K and used to investigate the tissue‐specific expression using the sequence‐specific primers listed in Table S3. The promoter fragment was then cloned into pDNOR222.1 and then inserted into pMDC164 to produce Pro_*PagFBL1*_::GUS constructs using the Gateway cloning system (Invitrogen) for transformation into poplar 84K via Agrobacteria (Shu *et al*., 2015). Derooted leafy stems of Pro_*PagFBL1*_::GUS lines were cultured on 1/2 MS for 0, 2, 3, 4, 5, 6 days, and the GUS staining was performed on the lower parts of stems. GUS staining during AR formation was performed as described by Shu (Shu *et al*., 2015). In brief, the samples were incubated in staining solution (20 mm X‐Gluc in phosphate buffer) for 12 h at 37 °C with gentle agitation at 70 r/min and then rinsed in 70% ethanol for visual observation and microscopy. Three replicates were included for each time point.

To explore the role of *PagFBL1* in AR formation in poplar 84K, *PagFBL1* cDNA from the cDNA of 84K was amplified using the sequence‐specific primers (Table S3), cloned into a plant overexpression vector pCAMBIA2301 (OE construct) and a binary pBI121 vector with antisense orientation (KD construct), respectively, as described in previous studies (Tang *et al*., 2010; Zhao *et al*., 2013), and transformed into poplar clone 84K. After obtaining the regenerated buds, we first induced their rooting using the screening medium (1/2 × MS) with the vector‐specific antibiotic (Hygromycin) and Timentin (for inhibiting Agrobacteria). The rooted transgenic plants were verified by genomic PCR and propagated by cutting. Then the expression level of *FBL1* in these transgenic lines was determined by qRT‐PCR, and the lines with intermediate change in expression level were selected in the following experiments. More than 19 lines have been generated for both OE and KD construct and eight OE lines (#4, #5, #7, #11, #15, #16, #17 and #18) and eight KD lines (#2, #5, #8, #10, #11, #12, #16 and #19) with intermediate *FBL1* expression levels in OE or KD lines were used for the experiments in this study. The root induction from leafy stems was performed on the OE, KD transgenic plants and 84K controls (WT) in 1/2 ×  MS at 24 ± 1 °C under cool‐white light (60 ± 5 μmol photons m^2^/s at the top leaves surface, 16‐h light/8‐h dark) (Shu *et al*., 2015), and the plants were checked and photographed after 96, 102, 108, 114, 120, 126, 138 h and 10 days. In addition, leaf explants for the OE transgenic plants (#4 and #18) and WT from 3‐week‐old seedlings were cultured on 1/2 × MS medium with sucrose in 0 μm and 5 μm IAA for *de novo* regeneration of ARs; leaf explants for the KD transgenic plants (#2 and #12) and WT from 3‐week‐old seedlings were cultured on 1/2 × MS medium with sucrose in 0 μm and 10 μm IAA for *de novo* regeneration of ARs. The cultured leaves were photographed after 11, 12, 14, 16 days. The OE transgenic plants (#4 and #18) and the KD transgenic plants (#2 and #12) were propagated by cuttings in soil as previously described (Shu *et al*., 2015) and grown for 5 months (OE #4, #18 and WT) and 2 months (KD #2, #12 and WT) in a glasshouse at Chinese Academy of Forestry. The ARs from the transgenic plants and WT were collected and measured. The experiments were performed with at least 30 clonal plants for each line.

### Plant phenotypic determination

The numbers of ARs were directly counted as described by Song and Xu (2011) to survey the difference in emerged and outgrown roots among the plant materials. Roots from cuttings grown on TS1 (Klasmann Deilmann, Germany) at 24 ± 1 °C with well‐watered and natural light from April to August in the glasshouse (Chinese Academy of Forestry, Beijing) were scanned using a root analysis machine (WinRhizoV4.0b; Regent instrument Inc., Quebec, Canada), and then, the roots were dried in an oven at 105 °C to a constant weight for measuring the root biomass. The measurements were performed on six individual plants for each line, and their mean and standard error were calculated (Data analysis).

### Sequence annotation and differential expression analysis

Leafy stems from 1‐month‐old seedlings (WT and #18) were subcultured into 1/2 × MS media. The bases of the stems (lowest 0.5 cm portion of the stem) were sampled at 0, 12, 24, 48 h during AR induction, frozen immediately in liquid nitrogen and stored at −80 °C before use. Three replicates were analysed consisting of about 100 stem segments for each. The RNAs were extracted using the RNeasy Plant Mini Kit and treated with RNase‐free DNase I (Qiagen, Hilden, Germany). RNA quality and quantity were determined using NanoDrop 1000 spectrophotometer (Thermo Fisher Scientific, Wilmington, DE). The clustering of the index‐coded samples was performed on a cBot Cluster Generation System using TruSeq PE Cluster Kit v4‐cBot‐HS (Illumia) according to the manufacturer's instructions, generating 2 × 150 bp and 1 × 60 bp reads. After cluster generation, the libraries were prepared and sequenced on an Illumina Hiseq 2500 platform. Image analysis and base calling were performed using the HiSeq Control Software version 1.4, and the Off‐Line Base Caller v1.9 ~ 120 million high quality RNA‐Seq reads (with quality score > 30 for each base) were pooled from Illumina sequencing of each of the 24 samples (three biological replicates of four stages) and were then assembled into contigs using Trinity. The paired‐end reads were generated by Biomarker Technologies (Fan *et al*., 2015). Gene function was annotated based on the following databases: Nr (ftp://ftp.ncbi.nih.gov/blast/db/); COG (http://www.ncbi.nlm.nih.gov/COG/); Swiss‐Prot (http://www.uniprot.org/); GO (http://www.geneontology.org/); KEGG (http://www.genome.jp/kegg/); and KOG (http://www.ncbi.nlm.nih.gov/KOG/). Differential expression analysis of two conditions was performed using the DEGseq software package in which a MA‐plot‐based method coupled to a random sampling model (MARS) method was mainly used. This approach was supplemented by the likelihood ratio test (LRT), Fisher's exact test (FET) and the fold‐change threshold on MA‐plot (FC) method (Li *et al*., 2015). The resulting *P* values were adjusted using the Benjamini and Hochberg's approach for controlling the false discovery rate (Storey and Tibshirani, 2003). Genes with an adjusted *P* value <0.05 found by DEGseq were assigned as differentially expressed with three biological replicates (Anders and Huber, 2010). The RNA‐Seq data were deposited in SRA database of NCBI with accession number SRP101893.

### RNA isolation and qRT‐PCR

Total RNAs from above stem samples at 0, 12, 24, 48 h were extracted, and their quality and quantity were checked as previously described. First‐strand cDNA synthesis was carried out with approximately 3 μg RNA using Superscript III reverse transcription kit (Life Technologies, Carlsbad, CA) according to the manufacturer's instruction. The amplified fragments were confirmed using agarose gel electrophoresis. Real‐time qRT‐PCR was performed as described by Shu (Shu *et al*., 2015) using *PagUBQ* gene as an internal reference (Table S4). All primer sequences used in the qRT‐PCR were described in Table S5. To confirm their expression patterns, nine auxin signalling‐related genes were selected for qRT‐PCR.

### Bimolecular fluorescence complementation (BiFC) assay

BiFC assay was performed as previously described (Sparkes *et al*., 2006). Complementary DNAs of *PagFBL1* and *PagIAA*s, including *PagIAA7.1*,* PagIAA7.2*,* PagIAA9*,* PagIAA12.1*,* PagIAA16.1*,* PagIAA16.2*,* PagIAA16.3*,* PagIAA16.4*,* PagIAA19.1*,* PagIAA20.1*,* PagIAA27.1*,* PagIAA28.1*,* PagIAA28.2*,* PagIAA29.2* and *PagIAA29.3*, were amplified using the primers listed in Table S3 and cloned into BiFC vectors pnYFP‐X for *PagFBL1* and pcCFP‐X for *PagIAA*s using the GATEWAY recombination system (Invitrogen). The pairs of constructs were cotransformed into leaves of 2‐month‐old tobacco (*Nicotiana benthamiana*) by infiltration as described previously (Shu *et al*., 2015). After 3 days, the leaves were treated with 0, 10, 100 μm, 1 mm NAA (Calderón Villalobos *et al*., 2012) (Sigma‐Aldrich, St. Louis, MO) and further incubated in a glasshouse for 3 days. The leaves were immersed in 50 μm DAPI (4′, 6‐diamidino‐2‐phenylindole), a nuclear marker, for 60 min. Fluorescence was observed using an UltraVIEW VoX 3D Live Cell Imaging System (PerkinElmer). For Confocal imaging YFP and DAPI fluorescence, 488 and 405‐nm laser and a 488 and 405‐nm band‐pass emission filter were used, respectively (Shu *et al*., 2015). The experiments were performed on three tobacco leaves for each pair of constructs and repeated three times.

### LexA yeast two‐hybrid assays

*FBL1* and *Aux/IAA* coding regions were cloned into the Y2H bait vector pGILDA and the prey vector pB42AD (Clontech), respectively, after amplifying using the primer pairs shown in Table S3. Bait and pray constructs were cotransformed into *Saccharomyces cerevisiae* strain EGY48[p8opLacZ] (Clontech), and transformants were selected on SD supplemented with –Ura/–His/–Trp dropout solution (BD Biosciences) and glucose medium. To test the interaction between FBL1 and Aux/IAA proteins, transformed yeast colonies were plated on SD‐galactose/raffinose‐inducing medium containing –Ura/–His/–Trp dropout supplement, 80 μg/mL X‐Gal and NAA in the different concentrations of 0 μm, 1 μm, 10 μm, 100 μm and incubated for 3‐4 days at 30 °C (Calderón Villalobos *et al*., 2012; Yu *et al*., 2015).

### Data analysis

Data were analysed by ANOVA using the SPSS 10 program (SPSS Inc., Chicago, IL). All data in the figures are given as means ± SE. Significance of differences between means was analysed by the two‐sample *t*‐test at *P *<* *0.05 or *P *<* *0.01. Asterisks on the histograms or after the mean value between the transgenic and WT, or among different treatments indicate they are statistically different.

## Supporting information


**Figure S1** AR formation in 8 *PagFBL1* OE and KD lines. (a) Expression of *FBL1* by qRT‐PCR analysis respectively. (b, c) AR rooting rates from leafy stems of 8 OE lines. (d, e) AR rooting rates of 8 KD lines. Bars = 1 cm. The values are means ± SE of 3 replicates. Significant differences between WT and transgenic lines are indicated with asterisks (*P <* *0.05 and **P <* *0.01).
**Figure S2** ARs from leaves of WT, *PagFBL1* OE lines #4 and #18 and KD lines #2 and #12 treated with (d, e, h) or without (b, c, g) IAA. (b, d) The induction rates of AR of WT and OE lines accessed during 11 and 16 days. (a, c, e) The number of ARs counted after 1 month for OE and WT lines. (f, g, h) ARs induction rate of WT and KD lines treated with or without IAA (10 μm). Bars = 5 mm. The values are means ± SE of 3 replicates. Significant differences between WT and transgenic lines are indicated with asterisks (*P <* *0.05 and **P <* *0.01).
**Figure S3** COG classification of DEGs. (a) 12 h vs 0 h in WT. (b) 24 h vs 12 h in WT. (c) 48 h vs 24 h in WT. (d) 12 h vs 0 h in #18. (e) 24 h vs 12 h in #18. (f) 48 h vs 24 h in #18.
**Figure S4** KEGG pathway of DEGs. (a) 12 h vs 0 h in WT. (b) 24 h vs 12 h in WT. (c) 48 h vs 24 h in WT. (d) 12 h vs 0 h in #18. (e) 24 h vs 12 h in #18. (f) 48 h vs 24 h in #18.
**Figure S5** Heat map showing the expression patterns of *PagIAAs* during AR formation from OE transgenic line (#18) and WT at four time points.
**Figure S6** The expressions of 15 *PagIAAs* during AR induction by qRT‐PCR.
**Figure S7** Interaction between PagFBL1 and PagIAA7.1, 7.2, 9, 12.1, 16.1, 16.2, 16.3, 16.4, 19.1, 20.1, 27.1, 29.2 or 29.3 with 100 μm NAA, respectively. Bars = 12 μm.
**Table S1** The same up‐regulated DEGs appeared from 0 h to 12 h after AR induction in #18 and from 12 h to 24 h after AR induction in WT.
**Table S2** Up‐regulated and down‐regulated genes of auxin signaling pathways related to AR induction at different time points.
**Table S3** The primer sequences for PCR amplification.
**Table S4** The stability of reference genes evaluated by different algorithms.
**Table S5** The primer sequences used in real‐time quantitative PCR.
