# Supplementary material for: The auxin receptor TIR1 homolog (PagFBL 1) regulates adventitious rooting through interactions with Aux/IAA28 in Populus
Source: Plant Biotechnol J. 2018 Jul 24;17(2):338–49. doi: 10.1111/pbi.12980 (PMC6335065; doi:10.1111/pbi.12980)
Supplement: Supplementary file 1 — Figure S1 AR formation in 8 PagFBL1 OE and KD lines. (a) Expression of FBL1 by qRT‐PCR analysis respectively. (b, c) AR rooting rates from leafy stems of 8 OE lines. (d, e) AR rooting rates of 8 KD lines. Bars = 1 cm. The values are means ± SE of 3 replicates. Significant differences between WT and transgenic lines are indicated with asterisks (*P < 0.05 and **P < 0.01). Figure S2 ARs from leaves of WT, PagFBL1 OE lines #4 and #18 and KD lines #2 and #12 treated with (d, e, h) or without (b, c, g) IAA. (b, d) The induction rates of AR of WT and OE lines accessed during 11 and 16 days. (a, c, e) The number of ARs counted after 1 month for OE and WT lines. (f, g, h) ARs induction rate of WT and KD lines treated with or without IAA (10 μm). Bars = 5 mm. The values are means ± SE of 3 replicates. Significant differences between WT and transgenic lines are indicated with asterisks (*P < 0.05 and **P < 0.01). Figure S3 COG classification of DEGs. (a) 12 h vs 0 h in WT. (b) 24 h vs 12 h in WT. (c) 48 h vs 24 h in WT. (d) 12 h vs 0 h in #18. (e) 24 h vs 12 h in #18. (f) 48 h vs 24 h in #18. Figure S4 KEGG pathway of DEGs. (a) 12 h vs 0 h in WT. (b) 24 h vs 12 h in WT. (c) 48 h vs 24 h in WT. (d) 12 h vs 0 h in #18. (e) 24 h vs 12 h in #18. (f) 48 h vs 24 h in #18. Figure S5 Heat map showing the expression patterns of PagIAAs during AR formation from OE transgenic line (#18) and WT at four time points. Figure S6 The expressions of 15 PagIAAs during AR induction by qRT‐PCR. Figure S7 Interaction between PagFBL1 and PagIAA7.1, 7.2, 9, 12.1, 16.1, 16.2, 16.3, 16.4, 19.1, 20.1, 27.1, 29.2 or 29.3 with 100 μm NAA, respectively. Bars = 12 μm. Table S1 The same up‐regulated DEGs appeared from 0 h to 12 h after AR induction in #18 and from 12 h to 24 h after AR induction in WT. Table S2 Up‐regulated and down‐regulated genes of auxin signaling pathways related to AR induction at different time points. Table S3 The primer sequences for PCR amplification. Table S4 The stability of refe [file PBI-17-338-s001.pdf]

The auxin receptor TIR1 homolog (PagFBL1) regulates adventitious rooting through interactions with Aux/IAA28 in *Populus*

Wenbo Shu, Houjun Zhou, Cheng Jiang, Shutang Zhao, Liuqiang Wang, Quanzi Li, Zhangqi Yang, Andrew Groover and Meng-Zhu Lu*

Additional material

Table S1. The same up-regulated DEGs appeared from 0 h to 12 h after AR induction in #18 and from 12 h to 24 h after AR induction in WT.

Gene_ID	#18-12h_vs_#18-0h_log2FC	#18-24h_vs_#18-12h_log2FC	WT-12h_vs_WT-0h_log2FC	WT-24h_vs_WT-12h_log2FC	COG_class_annotation	KOG_class	KEGG_pathway_annotation	Swissprot_annotation	nr_annotation
Potri.001G019900	Inf	0.2345810	Inf	1.8625550	--	--	--	Extensin-2 (Precursor) GN=T14E10_160 OS=Arabidopsis thaliana (Mouse-ear cress) PE=2 SV=1	hypothetical protein POPTR_0001s05740g [Populus trichocarpa]
Potri.001G020000	Inf	0.4696441	Inf	2.8966146	--	--	--	Extensin-2 (Precursor) GN=T14E10_160 OS=Arabidopsis thaliana (Mouse-ear cress) PE=2 SV=1	hypothetical protein POPTR_0001s05740g [Populus trichocarpa]
Potri.001G316000	1.8631920	0.9981807	0.7500300	1.2398488	--	--	--	--	PREDICTED: uncharacterized protein LOC105140484 isoform X3 [Populus euphratica]
Potri.001G391300	Inf	0.7302930	--	2.5266726	--	--	--	Epidermis-specific secreted glycoprotein EP1 (Precursor) GN=EP1 OS=Daucus carota (Wild carrot) PE=1 SV=1	PREDICTED: epidermis-specific secreted glycoprotein EP1-like [Populus euphratica]
Potri.003G134500	1.5267398	0.8153657	0.6928093	1.1518316	--	--	--	3-oxo-Delta(4,5)-steroid 5-beta-reductase GN=T22A6.50 OS=Arabidopsis thaliana (Mouse-ear cress) PE=1 SV=1	hypothetical protein POPTR_0003s13440g [Populus trichocarpa]
Potri.004G235400	2.3785447	0.4066500	2.0886848	1.5028184	Secondary metabolites biosynthesis, transport and catabolism	[QI]	Carotenoid biosynthesis (ko00906)	Abscisic acid 8'-hydroxylase 1 GN=CYP707A1 OS=Arabidopsis thaliana (Mouse-ear cress) PE=2 SV=1	cytochrome P450 family protein [Populus trichocarpa]
Potri.006G022800	Inf	-0.1472222	Inf	2.1324767	--	[GC]	--	7-deoxyloganetin glucosyltransferase GN=UGT85A24 OS=Gardenia jasminoides (Cape jasmine) PE=1 SV=1	hypothetical protein POPTR_0006s02390g [Populus trichocarpa]
Potri.007G049900	Inf	0.7804099	2.7937453	2.3872208	Secondary metabolites biosynthesis, transport and catabolism	[Q]	--	Isoflavone 2'-hydroxylase GN=CYP81E1 OS=Glycyrrhiza echinata (Licorice) PE=1 SV=2	cytochrome P450 family protein [Populus trichocarpa]

Potri.010G142900	Inf	0.7025578	-0.4393843	1.8985202	--	--	--	Basic leucine zipper 1 GN=BZIP1 OS=Arabidopsis thaliana (Mouse-ear cress) PE=1 SV=1	bZIP family transcription factor family protein [Populus trichocarpa]
Potri.011G110000	Inf	0.8553482	0.1534412	2.7545114	--	--	--	Epidermis-specific secreted glycoprotein EP1 (Precursor) GN=EP1 OS=Daucus carota (Wild carrot) PE=1 SV=1	curculin-like lectin family protein [Populus trichocarpa]
Potri.011G110500	2.1935498	0.5561984	-0.0425798	1.2733573	--	--	--	Epidermis-specific secreted glycoprotein EP1 (Precursor) GN=EP1 OS=Daucus carota (Wild carrot) PE=1 SV=1	curculin-like lectin family protein [Populus trichocarpa]
Potri.011G149400	4.2559801	1.1075760	--	2.1652676	--	--	--	--	hypothetical protein POPTR_0011s15240g [Populus trichocarpa]
Potri.011G155900	Inf	0.4596266	4.0697413	2.2121502	Energy production and conversion	--	--	Reticuline oxidase-like protein (Precursor) GN=At4g20830 OS=Arabidopsis thaliana (Mouse-ear cress) PE=1 SV=2	FAD-binding domain-containing family protein [Populus trichocarpa]
Potri.012G056800	Inf	0.4314388	Inf	2.9234351	--	--	--	LOB domain-containing protein 41 GN=F16B3.18 OS=Arabidopsis thaliana (Mouse-ear cress) PE=2 SV=1	hypothetical protein POPTR_0012s05340g [Populus trichocarpa]
Potri.014G145100	4.3826091	0.7344635	0.5754231	2.3999154	--	--	Flavonoid biosynthesis (ko00941);; Circadian rhythm - plant (ko04712)	Chalcone synthase GN=CHS OS=Vitis vinifera (Grape) PE=2 SV=1	chalcone synthase family protein [Populus trichocarpa]
Potri.019G093700	Inf	0.4561178	Inf	1.7976403	--	[R]	Amino sugar and nucleotide sugar metabolism (ko00520)	Endochitinase PR4 (Precursor) GN=CHI4 OS=Phaseolus vulgaris (Kidney bean) PE=2 SV=1	chitinase family protein, partial [Populus trichocarpa]
Potri.019G116600	2.3827853	0.5128408	-1.3601712	2.2006538	--	--	--	--	unknown [Populus trichocarpa]
Potri.001G336200	Inf	0.0336794	3.7323139	1.3988079	--	--	--	--	hypothetical protein POPTR_0001s36270g [Populus trichocarpa]
Potri.001G391200	Inf	0.7034396	4.1334437	1.9414137	--	--	--	Epidermis-specific secreted glycoprotein EP1 (Precursor) GN=EP1 OS=Daucus carota (Wild	hypothetical protein POPTR_0001s40180g [Populus

								carrot) PE=1 SV=1	trichocarpa]
Potri.001G451100	2.0700257	0.8462961	1.6379293	1.5689263	--	[R]	Phenylpropanoid biosynthesis (ko00940)	Caffeic acid 3-O-methyltransferase 1 GN=OMT1 OS=Populus tremuloides (Quaking aspen) PE=1 SV=1	eugenol O-methyltransferase family protein [Populus trichocarpa]
Potri.002G025500	Inf	1.4885245	Inf	2.0003781	Secondary metabolites biosynthesis, transport and catabolism	[Q]	--	Cytochrome P450 83B1 GN=At4g31500 OS=Arabidopsis thaliana (Mouse-ear cress) PE=1 SV=1	hypothetical protein POPTR_0002s02730g [Populus trichocarpa]
Potri.003G087000	5.8950565	0.4463679	Inf	1.7075820	--	--	--	Expansin-like B1 (Precursor) OS=Oryza sativa subsp. japonica (Rice) PE=2 SV=2	hypothetical protein POPTR_0003s08530g [Populus trichocarpa]
Potri.003G176900	Inf	1.2943108	3.5284598	1.8326922	--	--	Flavonoid biosynthesis (ko00941);; Circadian rhythm - plant (ko04712)	Chalcone synthase 1 GN=CHS1 OS=Camellia sinensis (Tea) PE=2 SV=1	naregenin-chalcone synthase family protein [Populus trichocarpa]
Potri.004G027400	Inf	0.4390289	5.3262516	1.2964380	--	--	--	G-type lectin S-receptor-like serine/threonine-protein kinase At1g11330 (Precursor) GN=At1g11330 OS=Arabidopsis thaliana (Mouse-ear cress) PE=2 SV=3	hypothetical protein POPTR_0004s02660g [Populus trichocarpa]
Potri.004G129400	1.0078797	0.7678296	4.1129767	1.2313974	--	[S]	Taurine and hypotaurine metabolism (ko00430)	--	PREDICTED: 2-aminoethanethiol dioxygenase-like [Populus euphratica]
Potri.004G182000	5.3259395	0.9756544	2.8264327	1.5235248	General function prediction only	[R]	Amino sugar and nucleotide sugar metabolism (ko00520)	Endochitinase 1 (Precursor) GN=CHIA1 OS=Theobroma cacao (Cacao) PE=2 SV=1	chitinase family protein [Populus trichocarpa]
Potri.005G113700	4.1902886	0.7546907	2.2134780	1.1652438	General function prediction only	[QR]	Flavonoid biosynthesis (ko00941)	Naringenin,2-oxoglutarate 3-dioxygenase (Fragment) GN=AN3 OS=Petunia hybrida (Petunia) PE=1 SV=1	unknown [Populus trichocarpa]
Potri.005G113900	2.1253587	0.8233892	2.0636448	1.0595721	General function prediction only	[QR]	Flavonoid biosynthesis (ko00941)	Naringenin,2-oxoglutarate 3-dioxygenase (Fragment) GN=AN3 OS=Petunia hybrida (Petunia) PE=1 SV=1	hypothetical protein POPTR_0005s11600g [Populus trichocarpa]
Potri.005G2	1.7271806	0.7557508	1.7839176	1.3926089	--	[V]	Flavonoid biosynthesis	Dihydroflavonol-4-reductase GN=DFR OS=Vitis vinifera (Grape)	hypothetical protein POPTR_0005s25110g [Populus

29500							(ko00941)	PE=1 SV=1	trichocarpa]
Potri.006G054800	2.7495031	0.7509783	3.7744735	1.0523047	--	[GMW]	--	Probable glucuronoxylan glucuronosyltransferase F8H GN=F8H OS=Arabidopsis thaliana (Mouse-ear cress) PE=2 SV=1	exostosin family protein [Populus trichocarpa]
Potri.006G117500	3.0988778	0.4443124	3.3747034	2.1145921	--	--	--	Putative E3 ubiquitin-protein ligase RING1a GN=K9L2.3 OS=Arabidopsis thaliana (Mouse-ear cress) PE=1 SV=2	hypothetical protein POPTR_0006s11870g [Populus trichocarpa]
Potri.008G116500	6.7518969	0.5894595	3.1589102	1.2438379	--	--	Flavonoid biosynthesis (ko00941)	Leucoanthocyanidin reductase GN=LAR OS=Desmodium uncinatum (Spanish clover) PE=1 SV=1	hypothetical protein POPTR_0008s11540g [Populus trichocarpa]
Potri.009G106000	6.3219711	0.5539113	7.6940071	1.7652481	--	--	--	--	glycine-rich family protein [Populus trichocarpa]
Potri.010G055400	4.5246317	0.8859788	4.8137126	1.0871733	Carbohydrate transport and metabolism	[G]	Glycolysis / Gluconeogenesis (ko00010);; Carbon fixation in photosynthetic organisms (ko00710);; Carbon metabolism (ko01200);; Biosynthesis of amino acids (ko01230)	Glyceraldehyde-3-phosphate dehydrogenase GAPC1, cytosolic GN=T6K12.26 OS=Arabidopsis thaliana (Mouse-ear cress) PE=1 SV=2	glyceraldehyde 3-phosphate dehydrogenase family protein [Populus trichocarpa]
Potri.010G075200	Inf	0.7740902	Inf	1.8389202	--	--	--	Glu S.griseus protease inhibitor OS=Momordica charantia (Bitter gourd) PE=1 SV=1	hypothetical protein POPTR_0010s08600g [Populus trichocarpa]
Potri.011G158400	3.3867026	0.2877452	5.9055145	1.0293191	Energy production and conversion	--	--	Reticuline oxidase-like protein (Precursor) GN=At4g20830 OS=Arabidopsis thaliana (Mouse-ear cress) PE=1 SV=2	FAD-binding domain-containing family protein [Populus trichocarpa]
Potri.012G102700	Inf	0.6503972	Inf	1.2237175	--	--	Plant hormone signal transduction (ko04075)	Indole-3-acetic acid-induced protein ARG7 GN=ARG7 OS=Vigna radiata var. radiata (Mung bean) PE=2 SV=1	hypothetical protein POPTR_0012s10450g [Populus trichocarpa]
Potri.013G041600	4.5835364	0.7339965	2.3334849	1.0739383	--	[R]	--	Win-like protein (Precursor) GN=HEV1 OS=Hevea brasiliensis (Para rubber tree) PE=1 SV=2	HEV1.2 family protein [Populus trichocarpa]

Potri.013G065100	Inf	0.2600984	Inf	1.4084307	--	--	--	GDSL esterase/lipase 1 (Precursor) GN=GLIP1 OS=Arabidopsis thaliana (Mouse-ear cress) PE=1 SV=1	50 kDa family protein [Populus trichocarpa]
Potri.013G083600	10.8758233	0.7879303	5.8054838	1.1528834	--	--	Phenylalanine metabolism (ko00360);; Phenylpropanoid biosynthesis (ko00940)	Peroxidase 4 {ECO:0000250|UniProtKB:Q42578} (Precursor) OS=Vitis vinifera (Grape) PE=1 SV=1	hypothetical protein POPTR_0013s08130g [Populus trichocarpa]
Potri.013G098900	6.7995934	0.9084790	2.8509229	1.2049052	Function unknown	[R]	--	DNA-damage-repair/toleration protein DRT100 (Precursor) OS=Arabidopsis thaliana (Mouse-ear cress) PE=2 SV=2	PREDICTED: probable leucine-rich repeat receptor-like protein kinase At1g35710 [Populus euphratica]
Potri.014G126100	2.0219358	0.6140248	1.7448529	1.1093054	--	--	--	Ethylene-responsive transcription factor RAP2-3 OS=Arabidopsis thaliana (Mouse-ear cress) PE=1 SV=2	hypothetical protein POPTR_0014s12090g [Populus trichocarpa]
Potri.019G057800	2.4819992	0.6566285	3.6418687	1.3255992	--	--	--	Probable chalcone--flavonone isomerase 3 GN=K18I23.7 OS=Arabidopsis thaliana (Mouse-ear cress) PE=1 SV=1	hypothetical protein POPTR_0019s08610g [Populus trichocarpa]
Potri.019G093800	Inf	0.5319319	Inf	2.1450749	--	[R]	Amino sugar and nucleotide sugar metabolism (ko00520)	Endochitinase PR4 (Precursor) GN=CHI4 OS=Phaseolus vulgaris (Kidney bean) PE=2 SV=1	hypothetical protein POPTR_0019s12360g [Populus trichocarpa]
Potri.001G051500	Inf	1.8630759	Inf	2.1823663	--	--	Flavonoid biosynthesis (ko00941);; Circadian rhythm - plant (ko04712)	Chalcone synthase 1 GN=CHS1 OS=Camellia sinensis (Tea) PE=2 SV=1	naregenin-chalcone synthase family protein [Populus trichocarpa]
Potri.001G282100	Inf	1.9040586	Inf	1.9601873	--	[GC]	--	UDP-glycosyltransferase 87A2 GN=UGT87A2 OS=Arabidopsis thaliana (Mouse-ear cress) PE=1 SV=1	hypothetical protein POPTR_0001s28890g [Populus trichocarpa]
Potri.001G334800	Inf	1.0205569	2.5690344	1.3119131	--	[R]	--	Kelch repeat-containing protein At3g27220 GN=At3g27220 OS=Arabidopsis thaliana (Mouse-ear cress) PE=2 SV=1	hypothetical protein POPTR_0001s34240g [Populus trichocarpa]
Potri.002G070100	9.4406502	1.7498587	2.9346849	2.1364238	Cell wall/membrane/envelope	--	--	Extensin (Precursor) OS=Daucus carota (Wild carrot) PE=2 SV=1	PREDICTED: extensin-3 isoform X2 [Populus euphratica]

					biogenesis				
Potri.002G108400	1.3531099	1.1326605	1.3312523	1.5365030	--	--	--	Transcription factor bHLH93 GN=K21L13.16 OS=Arabidopsis thaliana (Mouse-ear cress) PE=1 SV=1	hypothetical protein POPTR_0002s10910g [Populus trichocarpa]
Potri.004G030700	2.6826523	1.0044137	2.5347575	1.6810266	--	[V]	Flavonoid biosynthesis (ko00941)	Anthocyanidin reductase GN=T13M11.8 OS=Arabidopsis thaliana (Mouse-ear cress) PE=1 SV=2	Leucoanthocyanidin reductase family protein [Populus trichocarpa]
Potri.004G041000	3.1196187	1.4307615	3.0130025	1.9229099	--	--	--	CBS domain-containing protein CBSX5 GN=CBSX5 OS=Arabidopsis thaliana (Mouse-ear cress) PE=2 SV=2	hypothetical protein POPTR_0004s04120g [Populus trichocarpa]
Potri.004G114300	3.8255664	1.7673162	3.5012075	1.3532828	--	--	--	Vegetative cell wall protein gp1 (Precursor) GN=GP1 OS=Chlamydomonas reinhardtii (Chlamydomonas smithii) PE=1 SV=1	hypothetical protein POPTR_0004s11300g [Populus trichocarpa]
Potri.005G135300	7.8024684	2.5573549	3.6297763	1.9034604	--	--	Phenylalanine metabolism (ko00360);; Phenylpropanoid biosynthesis (ko00940)	Peroxidase 10 (Precursor) GN=F14J22.19 OS=Arabidopsis thaliana (Mouse-ear cress) PE=1 SV=1	peroxidase family protein [Populus trichocarpa]
Potri.005G188300	Inf	1.2535870	Inf	1.8046078	--	--	--	Pathogen-related protein OS=Hordeum vulgare (Barley) PE=2 SV=2	hypothetical protein POPTR_0005s21070g [Populus trichocarpa]
Potri.005G188400	Inf	1.0101648	Inf	1.4140165	--	--	--	Pathogen-related protein OS=Hordeum vulgare (Barley) PE=2 SV=2	putative pathogenesis-related family protein [Populus trichocarpa]
Potri.005G190100	Inf	1.8835820	3.4316353	2.6992261	Cell wall/membrane/envelope biogenesis	--	--	Extensin (Precursor) OS=Daucus carota (Wild carrot) PE=2 SV=1	PREDICTED: extensin isoform X4 [Populus euphratica]
Potri.008G174900	6.7838984	1.0575690	4.0313597	1.3199862	Posttranslational modification, protein turnover, chaperones	[O]	Glutathione metabolism (ko00480)	Glutathione transferase GST 23 OS=Zea mays (Maize) PE=2 SV=1	hypothetical protein POPTR_0008s17500g [Populus trichocarpa]
Potri.010G1	6.1888506	1.5620878	3.4633578	1.7749506	Carbohydrate transport and	--	--	Glucan endo-1,3-beta-glucosidase major form (Precursor) GN=HGN1	Glucan endo-1 family protein [Populus

42800					metabolism			OS=Hevea brasiliensis (Para rubber tree) PE=1 SV=2	trichocarpa]
Potri.010G208600	5.1610559	1.1122946	6.6409674	1.0074689	--	--	--	Transcription factor bHLH110 GN=T22C5.11 OS=Arabidopsis thaliana (Mouse-ear cress) PE=2 SV=2	PREDICTED: transcription factor bHLH123-like [Populus euphratica]
Potri.013G000500	4.3660183	1.6971185	4.8674382	1.6783155	--	--	--	Germin-like protein subfamily 2 member 1 (Precursor) GN=F14J9.22 OS=Arabidopsis thaliana (Mouse-ear cress) PE=2 SV=2	rhicadhesin receptor family protein [Populus trichocarpa]
Potri.014G019200	2.9214638	1.5888717	1.7113102	2.2279907	--	[C]	--	Cytochrome b5 OS=Nicotiana tabacum (Common tobacco) PE=2 SV=1	hypothetical protein POPTR_0014s01940g [Populus trichocarpa]
Potri.014G108800	6.7431254	1.2725427	7.4405966	1.8939204	--	--	--	--	hypothetical protein POPTR_0014s10440g [Populus trichocarpa]
Potri.014G128200	1.2271486	1.1407659	1.7116595	1.4705985	--	[KLO]	Base excision repair (ko03410)	Poly [ADP-ribose] polymerase 2 GN=T14P8.19 OS=Arabidopsis thaliana (Mouse-ear cress) PE=2 SV=1	Poly [ADP-ribose] polymerase family protein [Populus trichocarpa]
Potri.016G102100	5.1647480	1.3135997	7.4741765	1.0095374	General function prediction only	[R]	--	Aldo-keto reductase family 4 member C9 GN=AKR4C9 OS=Arabidopsis thaliana (Mouse-ear cress) PE=1 SV=1	hypothetical protein POPTR_0016s10390g [Populus trichocarpa]
Potri.016G132700	10.3045469	1.1286335	6.3718147	1.6302065	--	--	Phenylalanine metabolism (ko00360);; Phenylpropanoid biosynthesis (ko00940)	Cationic peroxidase 1 (Precursor) GN=PNC1 OS=Arachis hypogaea (Peanut) PE=1 SV=2	hypothetical protein POPTR_0016s14030g [Populus trichocarpa]
Potri.017G140900	6.5706621	1.7919022	2.4855520	2.2557816	--	--	Glutathione metabolism (ko00480)	--	hypothetical protein POPTR_0017s01880g [Populus trichocarpa]
Potri.017G151900	3.3297647	1.1966605	3.7053592	1.4858123	--	[EH]	Glycolysis / Gluconeogenesis (ko00010)	Pyruvate decarboxylase 1 GN=PDC1 OS=Pisum sativum (Garden pea) PE=2 SV=1	hypothetical protein POPTR_0017s00800g [Populus trichocarpa]
Potri.018G1	Inf	1.8141010	3.7047991	1.6032880	--	[G]	Galactose metabolism (ko00052);; Glycerolipid metabolism (ko00561);;	Alpha-galactosidase (Precursor) OS=Coffea arabica (Arabian coffee)	hypothetical protein POPTR_0018s14920g [Populus

52200							Sphingolipid metabolism (ko00600);; Glycosphingolipid biosynthesis - globo series (ko00603)	PE=1 SV=1	trichocarpa]
Potri.019G128600	1.4512704	1.3981207	1.4852795	1.0124916	--	[R]	--	Probable serine/threonine-protein kinase WNK4 GN=WNK4 OS=Arabidopsis thaliana (Mouse-ear cress) PE=1 SV=1	hypothetical protein POPTR_0019s14910g [Populus trichocarpa]
Potri.T021700	3.0955456	1.5511470	Inf	2.0185994	--	--	--	G-type lectin S-receptor-like serine/threonine-protein kinase At4g27290 (Precursor) GN=At4g27290 OS=Arabidopsis thaliana (Mouse-ear cress) PE=3 SV=4	hypothetical protein POPTR_0011s12970g [Populus trichocarpa]
Potri.T167100	6.1926682	1.4499330	3.2240538	1.7643513	--	--	--	Glucan endo-1,3-beta-glucosidase major form (Precursor) GN=HGN1 OS=Hevea brasiliensis (Para rubber tree) PE=1 SV=2	hypothetical protein POPTR_0010s15320g [Populus trichocarpa]
--	Inf	1.8963181	--	3.2227618	--	--	--	--	Uncharacterized protein isoform 3, partial [Theobroma cacao]
--	4.7596084	1.4590402	2.3294568	1.6405161	--	--	--	--	--
Potri.001G019700	7.5811629	2.4824420	3.0581332	3.6503889	--	--	--	Extensin (Precursor) OS=Daucus carota (Wild carrot) PE=2 SV=1	hypothetical protein POPTR_0001s05720g [Populus trichocarpa]
Potri.001G097300	4.4568074	3.6672232	-0.1634970	4.0998106	--	[R]	--	Aluminum-activated malate transporter 9 GN=ALMT9 OS=Arabidopsis thaliana (Mouse-ear cress) PE=2 SV=1	hypothetical protein POPTR_0001s10080g [Populus trichocarpa]
Potri.001G102400	4.8782026	1.4344343	1.3596338	1.8574515	--	--	--	Osmotin-like protein OSM34 (Precursor) GN=OSM34 OS=Arabidopsis thaliana (Mouse-ear cress) PE=2 SV=2	hypothetical protein POPTR_0001s09570g [Populus trichocarpa]
Potri.001G107600	3.2056890	1.7841821	0.8852815	1.8342008	--	--	--	Osmotin-like protein OSM34 (Precursor) GN=OSM34 OS=Arabidopsis thaliana (Mouse-ear cress) PE=2 SV=2	P21 family protein [Populus trichocarpa]

Potri.001G107800	Inf	3.3747407	--	5.2887831	--	--	--	Thaumatin-like protein (Precursor) GN=tlp OS=Actinidia deliciosa (Kiwi) PE=1 SV=2	P21 family protein [Populus trichocarpa]
Potri.001G140700	Inf	1.8403397	2.4640513	2.5237492	--	[V]	--	Cinnamoyl-CoA reductase 1 GN=T24D18.5 OS=Arabidopsis thaliana (Mouse-ear cress) PE=1 SV=1	hypothetical protein POPTR_0001s02320g [Populus trichocarpa]
Potri.001G250800	1.6295795	1.5447246	-0.1558234	1.6980408	--	[O]	--	RING-H2 finger protein ATL66 GN=ATL66 OS=Arabidopsis thaliana (Mouse-ear cress) PE=2 SV=1	hypothetical protein POPTR_0001s25790g [Populus trichocarpa]
Potri.001G270800	3.8345671	2.0047061	Inf	3.6836019	Secondary metabolites biosynthesis, transport and catabolism	[QI]	--	Cytochrome P450 87A3 GN=OSJNBa0088I22.16 OS=Oryza sativa subsp. japonica (Rice) PE=2 SV=3	hypothetical protein POPTR_0001s27770g [Populus trichocarpa]
Potri.001G288600	Inf	1.8972541	-0.2261867	1.8092069	Function unknown	[S]	Plant hormone signal transduction (ko04075);; Plant-pathogen interaction (ko04626)	Pathogenesis-related protein 1 (Precursor) GN=At2g14610 OS=Arabidopsis thaliana (Mouse-ear cress) PE=1 SV=1	Pathogenesis-related family protein [Populus trichocarpa]
Potri.003G119100	Inf	1.9252558	1.5631216	2.6126004	--	[QR]	Flavonoid biosynthesis (ko00941)	Leucoanthocyanidin dioxygenase GN=ANS OS=Malus domestica (Apple) PE=2 SV=1	Leucoanthocyanidin dioxygenase family protein [Populus trichocarpa]
Potri.003G147600	4.0527738	2.7040455	--	2.6895071	--	--	--	Endoglucanase 1 (Precursor) GN=CEL1 OS=Persea americana (Avocado) PE=2 SV=1	endo-1,4-beta-glucanase [Populus trichocarpa]
Potri.004G000400	Inf	2.5978676	--	4.9636728	--	--	--	Miraculin (Precursor) OS=Synsepalum dulcificum (Miracle fruit) PE=1 SV=3	truncated Kunitz trypsin inhibitor family protein [Populus trichocarpa]
Potri.004G054100	1.9988037	1.1591173	0.2063910	1.5157118	--	[EH]	Glycolysis / Gluconeogenesis (ko00010)	Pyruvate decarboxylase 1 GN=PDC1 OS=Pisum sativum (Garden pea) PE=2 SV=1	pyruvate decarboxylase family protein [Populus trichocarpa]
Potri.004G129700	3.0490893	1.2349875	0.4364916	1.4607174	Carbohydrate transport and metabolism	[G]	Glycolysis / Gluconeogenesis (ko00010);; Galactose metabolism (ko00052)	--	hypothetical protein POPTR_0004s13470g [Populus trichocarpa]

Potri.004G136000	Inf	2.3803442	-0.1446310	3.3745880	--	--	--	Dehydration-responsive protein RD22 (Precursor) GN=RD22 OS=Arabidopsis thaliana (Mouse-ear cress) PE=2 SV=1	hypothetical protein POPTR_0129s00200g [Populus trichocarpa]
Potri.004G233500	Inf	1.7203601	1.7909313	1.8871679	--	[R]	--	Hexose carrier protein HEX6 GN=HEX6 OS=Ricinus communis (Castor bean) PE=2 SV=1	hypothetical protein POPTR_0004s24170g [Populus trichocarpa]
Potri.005G048400	1.0980868	1.5700669	1.6561078	1.5487506	Amino acid transport and metabolism	[E]	Cysteine and methionine metabolism (ko00270);; Sulfur metabolism (ko00920);; Carbon metabolism (ko01200);; Biosynthesis of amino acids (ko01230)	Cysteine synthase OS=Citrullus lanatus (Watermelon) PE=1 SV=1	O-acetylserine (thiol)lyase family protein [Populus trichocarpa]
Potri.005G203200	5.1747737	1.3685843	0.5757906	1.1310831	--	--	--	Probable WRKY transcription factor 28 GN=WRKY28 OS=Arabidopsis thaliana (Mouse-ear cress) PE=2 SV=1	hypothetical protein POPTR_0005s22480g [Populus trichocarpa]
Potri.006G212000	Inf	1.4852158	--	3.2699240	--	--	--	Inhibitor of trypsin and hageman factor OS=Cucurbita maxima (Pumpkin) PE=1 SV=1	hypothetical protein POPTR_0006s22860g [Populus trichocarpa]
Potri.007G103400	Inf	1.2467986	--	1.8412255	--	[R]	--	--	hypothetical protein POPTR_0007s04400g [Populus trichocarpa]
Potri.007G111600	Inf	1.8468562	--	2.4487250	--	--	--	Alpha-amylase/subtilisin inhibitor (Precursor) GN=OSJNBa0038O10.14 OS=Oryza sativa subsp. japonica (Rice) PE=1 SV=2	hypothetical protein POPTR_0007s03640g [Populus trichocarpa]
Potri.008G106400	5.6029566	1.8493370	2.3001769	2.7049131	--	[R]	alpha-Linolenic acid metabolism (ko00592)	Alpha-dioxygenase 1 (Precursor) GN=T13O15.6 OS=Arabidopsis thaliana (Mouse-ear cress) PE=1 SV=1	putative alpha-dioxygenase family protein [Populus trichocarpa]
Potri.009G064900	Inf	1.2093849	Inf	1.9113764	Secondary metabolites biosynthesis, transport and catabolism	[QI]	--	Cytochrome P450 87A3 GN=OSJNBa0088I22.16 OS=Oryza sativa subsp. japonica (Rice) PE=2 SV=3	PREDICTED: cytochrome P450 87A3-like [Populus euphratica]

Potri.011G025400	Inf	3.6142033	1.3262892	3.1790549	--	--	Pentose and glucuronate interconversions (ko00040);; Starch and sucrose metabolism (ko00500)	Bifunctional pectinesterase 18/rRNA N-glycosylase (Precursor) OS=Arabidopsis thaliana (Mouse-ear cress) PE=1 SV=3	pectin methylesterase family protein [Populus trichocarpa]
Potri.011G158100	Inf	3.5975362	--	4.5316914	Energy production and conversion	--	--	Cannabidiolic acid synthase-like 1 (Precursor) GN=CBDAS2 OS=Cannabis sativa (Hemp) PE=2 SV=1	PREDICTED: cannabidiolic acid synthase-like 1 [Populus euphratica]
Potri.011G162800	2.2602463	1.7339461	2.6177524	2.2201507	Energy production and conversion	--	--	Tetrahydrocannabinolic acid synthase (Precursor) OS=Cannabis sativa (Hemp) PE=1 SV=1	hypothetical protein POPTR_0011s16560g [Populus trichocarpa]
Potri.012G047300	2.1617667	2.0094697	2.2743760	2.1312926	--	[IR]	Glycerophospholipid metabolism (ko00564)	Phosphomethylethanolamine N-methyltransferase OS=Arabidopsis thaliana (Mouse-ear cress) PE=2 SV=2	hypothetical protein POPTR_0012s044902g, partial [Populus trichocarpa]
Potri.013G154400	Inf	1.9471322	7.2189038	2.9804165	--	--	Phenylalanine metabolism (ko00360);; Phenylpropanoid biosynthesis (ko00940)	Cationic peroxidase 1 (Precursor) GN=PNC1 OS=Arachis hypogaea (Peanut) PE=1 SV=2	peroxidase family protein [Populus trichocarpa]
Potri.014G118400	3.0759103	1.7476758	1.5252334	2.4815588	--	--	Galactose metabolism (ko00052)	Stachyose synthase (Precursor) GN=STS1 OS=Pisum sativum (Garden pea) PE=1 SV=1	stachyose synthase family protein [Populus trichocarpa]
Potri.015G006000	2.8760124	1.4214324	0.4292026	1.7876111	Defense mechanisms	[Q]	--	Pleiotropic drug resistance protein 3 GN=PDR3 OS=Nicotiana tabacum (Common tobacco) PE=2 SV=1	hypothetical protein POPTR_0015s00840g [Populus trichocarpa]
Potri.015G120900	Inf	1.9087612	Inf	2.7313057	--	[I]	Steroid biosynthesis (ko00100);; Sesquiterpenoid and triterpenoid biosynthesis (ko00909)	Squalene monooxygenase OS=Panax ginseng (Korean ginseng) PE=2 SV=1	hypothetical protein POPTR_0015s15430g [Populus trichocarpa]
Potri.016G057400	Inf	2.1152896	2.3229854	2.9381059	--	--	--	Glucan endo-1,3-beta-glucosidase, basic isoform (Precursor) GN=GNS1 OS=Prunus persica (Peach) PE=3 SV=1	hypothetical protein POPTR_0016s05780g [Populus trichocarpa]
Potri.016G079100	9.1656480	1.1056528	Inf	2.0341103	--	--	--	Inhibitor of trypsin and hageman factor OS=Cucurbita maxima (Pumpkin) PE=1 SV=1	hypothetical protein POPTR_0016s07990g [Populus trichocarpa]

Potri.018G066900	3.5107343	3.0288962	2.0638928	3.7253336	--	[R]	--	Transcription initiation factor TFIID subunit 15 GN=TAF15 OS=Arabidopsis thaliana (Mouse-ear cress) PE=1 SV=1	hypothetical protein POPTR_0018s06970g [Populus trichocarpa]
Potri.018G129400	Inf	2.8683207	--	4.2498704	--	--	--	Blue copper protein (Precursor) GN=F5O24.120 OS=Arabidopsis thaliana (Mouse-ear cress) PE=1 SV=2	hypothetical protein POPTR_0018s12950g [Populus trichocarpa]
Potri.019G117500	Inf	1.1281051	--	4.4313875	--	--	--	--	hypothetical protein POPTR_0019s14010g [Populus trichocarpa]
Potri.T060100	2.9469202	1.3499023	-0.7622789	1.2915060	General function prediction only	[RV]	Pyruvate metabolism (ko00620)	Protein DJ-1 homolog D GN=DJ1D OS=Arabidopsis thaliana (Mouse-ear cress) PE=1 SV=1	hypothetical protein POPTR_0017s01530g [Populus trichocarpa]
Potri.T111200	Inf	1.6419463	--	3.4029646	--	--	--	Major allergen Pru ar 1 OS=Prunus armeniaca (Apricot) PE=1 SV=1	hypothetical protein POPTR_0016s04580g [Populus trichocarpa]
Potri.T163000	Inf	1.6190942	--	3.6084078	--	--	Phenylalanine metabolism (ko00360);; Phenylpropanoid biosynthesis (ko00940)	Lignin-forming anionic peroxidase (Precursor) OS=Nicotiana sylvestris (Wood tobacco) PE=2 SV=1	hypothetical protein POPTR_0010s24340g [Populus trichocarpa]
Potri.T175200	Inf	1.9411482	-0.3844577	2.0846357	General function prediction only	[R]	Amino sugar and nucleotide sugar metabolism (ko00520)	Endochitinase A2 (Precursor) GN=CHI2 OS=Pisum sativum (Garden pea) PE=1 SV=2	hypothetical protein POPTR_0790s00210g [Populus trichocarpa]

Table S2. Up-regulated and down-regulated genes of auxin signaling pathways related to AR induction at different time points.

Gene Name	Gene_ID	#18-12h_vs_#18-0h_log2FC	#18-24h_vs_#18-12h_log2FC	#18-48h_vs_#18-24h_log2FC	WT-12h_vs_WT-0h_log2FC	WT-24h_vs_WT-12h_log2FC	WT-48h_vs_WT-24h_log2FC	COG_class_annotation	KOG_class_annotation	Swissprot_annotation	nr_annotation
26S-4	Potri.015G007800	3.8075524	-0.3731043	-0.1846246	4.3794750	-0.0593056	-0.9108942	Posttranslational modification, protein turnover, chaperones	Posttranslational modification, protein turnover, chaperones	26S protease regulatory subunit 4 homolog GN=P0034A04.112 OS=Oryza sativa subsp. japonica (Rice) PE=2 SV=2	hypothetical protein POPTR_0015s01020g [Populus trichocarpa]
26S-6A	Potri.015G067400	2.7179564	-0.1835535	-0.6850570	2.9401010	-0.3820875	-0.3630234	Posttranslational modification, protein turnover, chaperones	Posttranslational modification, protein turnover, chaperones	26S protease regulatory subunit 6A homolog OS=Oryza sativa subsp. japonica (Rice) PE=2 SV=2	hypothetical protein POPTR_0015s07870g [Populus trichocarpa]
26S-6B	Potri.006G029100	2.2654202	0.1116214	0.1215391	2.3411286	-0.0064829	0.4270731	Posttranslational modification, protein turnover, chaperones	Posttranslational modification, protein turnover, chaperones	26S protease regulatory subunit 6B homolog {ECO:0000250|UniProtKB:P54778} OS=Helianthus annuus (Common sunflower) PE=1 SV=1	PREDICTED: 26S protease regulatory subunit 6B homolog [Populus euphratica]
26S-8	Potri.007G020800	4.1192802	-0.7251905	-0.7736124	1.8541792	-0.5536717	-0.9447426	Posttranslational modification, protein turnover, chaperones	Posttranslational modification, protein turnover, chaperones	26S protease regulatory subunit 8 homolog B GN=F28I16.150 OS=Arabidopsis thaliana (Mouse-ear cress) PE=1 SV=1	PREDICTED: uncharacterized AAA domain-containing protein C31G5.19-like [Populus euphratica]
ARF7.3	Potri.018G145800	1.0428527	-0.8961010	-0.1168651	1.6167895	-0.6898670	0.1283382	--	--	Auxin response factor 19 OS=Oryza sativa subsp. japonica (Rice) PE=2 SV=2	auxin response factor 2 family protein [Populus trichocarpa]
CUL1	Potri.008G224100	2.5184053	0.1812459	-0.3422988	2.0287016	0.1503999	-0.2933289	Posttranslational modification, protein turnover, chaperones	Cell cycle control, cell division, chromosome partitioning	Cullin-1 GN=CUL1 OS=Arabidopsis thaliana (Mouse-ear cress) PE=1 SV=1	hypothetical protein POPTR_0008s21900g [Populus trichocarpa]
GH3.1	Potri.007G050300	10.5739116	0.3960134	0.1710195	9.1831844	1.5729515	0.5824774	--	--	Probable indole-3-acetic acid-amido synthetase GH3.1 GN=GH3.1 OS=Arabidopsis thaliana (Mouse-ear cress) PE=2	hypothetical protein POPTR_0007s10350g [Populus trichocarpa]

										SV=1	
GH3.6	Potri.014G 136800	3.182275 2	0.2004431	-0.81265 00	1.9730494	0.0739825	-0.1110947	--	--	Probable indole-3-acetic acid-amido synthetase GH3.6 GN=OJ1607_F09.5 OS=Oryza sativa subsp. japonica (Rice) PE=2 SV=2	hypothetical protein POPTR_0014s13260g [Populus trichocarpa]
ARF5.2	Potri.005G 236700	3.047627 6	-0.2173386	-0.31448 79	-0.8075387	-0.2581722	-0.4974767	--	--	Auxin response factor 5 GN=F6F9.10 OS=Arabidopsis thaliana (Mouse-ear cress) PE=1 SV=3	PREDICTED: auxin response factor 5-like [Populus euphratica]
ARF5.1	Potri.002G 024700	1.599597 3	0.0117308	0.117889 4	1.4534820	-0.2245477	0.1929358	--	--	Auxin response factor 5 GN=F6F9.10 OS=Arabidopsis thaliana (Mouse-ear cress) PE=1 SV=3	hypothetical protein POPTR_0002s02630g [Populus trichocarpa]
P450-705A 5	Potri.001G 270900	2.860387 8	1.2931721	0.534060 0	0.7853223	0.9728606	0.7230789	Secondary metabolites biosynthesis, transport and catabolism	Secondary metabolites biosynthesis, transport and catabolism	Cytochrome P450 705A5 GN=MDN11.4 OS=Arabidopsis thaliana (Mouse-ear cress) PE=2 SV=1	hypothetical protein POPTR_0001s27780g [Populus trichocarpa]
P450-81D1 1	Potri.014G 020600	Inf	0.7894733	2.306420 5	1.2012081	0.7589166	0.8225278	Secondary metabolites biosynthesis, transport and catabolism	Secondary metabolites biosynthesis, transport and catabolism	Cytochrome P450 81D11 GN=CYP81D11 OS=Arabidopsis thaliana (Mouse-ear cress) PE=2 SV=1	hypothetical protein POPTR_0458s00200g [Populus trichocarpa]
P450-81E8	Potri.014G 020400	2.598106 5	-0.2523913	0.303546 2	4.2525020	-0.5865136	0.1966683	Secondary metabolites biosynthesis, transport and catabolism	Secondary metabolites biosynthesis, transport and catabolism	Cytochrome P450 81E8 {ECO:0000303|PubMed:146170 78} OS=Medicago truncatula (Barrel medic) PE=2 SV=1	cytochrome P450 family protein [Populus trichocarpa]
P450-82A3	Potri.014G 037400	Inf	0.8113303	0.542836 9	Inf	1.2243784	1.1363907	Secondary metabolites biosynthesis, transport and catabolism	Secondary metabolites biosynthesis, transport and catabolism	Cytochrome P450 82A3 GN=CYP82A3 OS=Glycine max (Soybean) PE=2 SV=1	hypothetical protein POPTR_0014s03700g [Populus trichocarpa]
P450-94A1	Potri.005G 220700	2.161943 1	-0.2921086	0.125900 0	1.5691581	-0.1872032	0.0099573	Secondary metabolites biosynthesis, transport and	--	Cytochrome P450 94A1 GN=CYP94A1 OS=Vicia sativa (Spring vetch) PE=2 SV=2	cytochrome P450 CYP94A1 family protein [Populus trichocarpa]

								catabolism			
E3-1	Potri.003G 129900	2.479503 7	-0.3792936	0.020798 4	0.7994301	-0.2620306	-0.1752268	--	Posttranslational modification, protein turnover, chaperones	E3 ubiquitin-protein ligase At3g02290 OS=Arabidopsis thaliana (Mouse-ear cress) PE=2 SV=1	hypothetical protein POPTR_0003s13000g [Populus trichocarpa]
E3-ATL41	Potri.019G 091400	Inf	-0.8653536	0.748718 4	Inf	-0.5988601	0.8086669	Posttranslational modification, protein turnover, chaperones	Posttranslational modification, protein turnover, chaperones	E3 ubiquitin-protein ligase ATL41 GN=ATL41 OS=Arabidopsis thaliana (Mouse-ear cress) PE=1 SV=1	PREDICTED: E3 ubiquitin-protein ligase ATL41-like [Populus euphratica]
E3-HOS1	Potri.001G 155200	4.585028 0	-0.2916073	3.324069 2	2.8085817	-0.2151066	1.2004735	--	--	E3 ubiquitin-protein ligase HOS1 GN=HOS1 OS=Arabidopsis thaliana (Mouse-ear cress) PE=1 SV=1	hypothetical protein POPTR_0001s15500g [Populus trichocarpa]
E3-PUB24	Potri.016G 069500	5.102268 1	0.9161986	0.243400 6	2.8021461	1.6191521	0.7862702	--	--	E3 ubiquitin-protein ligase PUB24 GN=PUB24 OS=Arabidopsis thaliana (Mouse-ear cress) PE=1 SV=1	U-box domain-containing family protein [Populus trichocarpa]
E3-RING1	Potri.009G 034800	1.528861 3	-0.7628189	0.404312 3	1.3859262	-0.5671285	0.4036926	--	Posttranslational modification, protein turnover, chaperones	E3 ubiquitin-protein ligase RING1 GN=RING1 OS=Gossypium hirsutum (Upland cotton) PE=1 SV=1	zinc finger family protein [Populus trichocarpa]
E3-XBAT3 2	Potri.018G 098400	1.204678 3	-0.5863591	-0.19428 31	1.5529142	-0.7184409	-0.1612685	General function prediction only	--	E3 ubiquitin-protein ligase XBAT32 GN=XBAT32 OS=Arabidopsis thaliana (Mouse-ear cress) PE=1 SV=1	hypothetical protein POPTR_0018s10750g [Populus trichocarpa]
E3-RHA2B	Potri.005G 081300	4.330057 8	-0.6439968	1.100584 7	0.4369660	-0.2758707	--	Posttranslational modification, protein turnover, chaperones	Posttranslational modification, protein turnover, chaperones	Probable E3 ubiquitin-protein ligase RHA2B GN=RHA2B OS=Arabidopsis thaliana (Mouse-ear cress) PE=1 SV=2	hypothetical protein POPTR_0005s08310g [Populus trichocarpa]
GH3.5	Potri.014G 095500	1.243205 1	0.4105617	0.017726 8	0.3763779	0.2784618	-0.1048236	--	--	Probable indole-3-acetic acid-amido synthetase GH3.5 GN=OSJNBa0009C07.16 OS=Oryza sativa subsp. japonica (Rice) PE=2 SV=1	hypothetical protein POPTR_0014s09120g [Populus trichocarpa]
IAA28.1	Potri.018G 057000	-0.76717 7141	-0.464928	-0.26247 7	-1.471666210 789773	-0.680148	-0.606031	--	--	Auxin-responsive protein IAA26 GN=MDC8.13 OS=Arabidopsis thaliana (Mouse-ear cress) PE=1	PREDICTED: auxin-responsive protein IAA2-like [Populus

										SV=2	euphratica]
IAA28.2	Potri.006G 236200	-1.20651 0202	0.289221	-0.62944 8988721 711	-1.236529	0.510157	-0.049081	--	--	Auxin-responsive protein IAA26 GN=MDC8.13 OS=Arabidopsis thaliana (Mouse-ear cress) PE=1 SV=2	hypothetical protein POPTR_0006s25250g [Populus trichocarpa]

Table S3. The primer sequences for PCR amplification.

Genes	Gene ID	Primer sequence F (5' to 3')	Primer sequence R (5' to 3')
Pro$_{PagFBL1}$::GUS	KY020444	GGGGACAACTTTGTACAAAAAAGTTGGATGGGCACGACGAAAACGGTTACCT	GGCGGCCGCACAACTTTGTACAAGAAAGTTGGGTAAACGAATTCGCCTTTCTCAA
PagFBL1-OE	KY020444	GGGGACAACTTTGTACAAAAAAGTTGGAATGTTGAGAAAGGCGAATTC	GGCGGCCGCACAACTTTGTACAAGAAAGTTGGGTACTAGGCCCCGCAATGGTTTCTG
PagFBL1-SE	KY020444	TGACAGAGTTGAGGGTGTTTCCATC	CTCCAACTTTTTGGCGTAAGTTCCA
PagIAA16.4	Potri.002G044900	GGGGACAACTTTGTACAAAAAAGTTGGAATGGAAGTGGAGAAGGGAACAA	GGCGGCCGCACAACTTTGTACAAGAAAGTTGGGTACTACTTGTAGCTTCTGTTCTTGCAC
PagIAA28.1	Potri.018G057000	GGGGACAACTTTGTACAAAAAAGTTGGAATGGAGGAATATATTCAGGGCTT	GGCGGCCGCACAACTTTGTACAAGAAAGTTGGGTATCATCTTCCGATTTCTACTGCAG
PagIAA28.2	Potri.006G236200	GGGGACAACTTTGTACAAAAAAGTTGGAATGGAGGAAAATACTCGGTGTTTC	GGCGGCCGCACAACTTTGTACAAGAAAGTTGGGTATCATCTTCCAAGTTCTACTGCACAA
PagIAA7.1	Potri.010G078300	GGGGACAACTTTGTACAAAAAAGTTGGAATGGCAACAGCTACCGTGTTAGGT	GGCGGCCGCACAACTTTGTACAAGAAAGTTGGGTATTACAGAAGCAAGCTGTTGTTAATT
PagIAA7.2	Potri.008G161200	GGGGACAACTTTGTACAAAAAAGTTGGAATGACAACTTCTGTGCTAGGTAC	GGCGGCCGCACAACTTTGTACAAGAAAGTTGGGTATCAGGTTCTGCTCTTGCATTTCTCC
PagIAA19.1	Potri.001G177500	GGGGACAACTTTGTACAAAAAAGTTGGAATGGCACAACCTCTTGGACTAG	GGCGGCCGCACAACTTTGTACAAGAAAGTTGGGTATCAGTCACGATCATCTTTTGAAATA
PagIAA20.1	Potri.002G186400	GGGGACAACTTTGTACAAAAAAGTTGGAATGGGCAGAGGAGCTGCCTCTA	GGCGGCCGCACAACTTTGTACAAGAAAGTTGGGTATCATAGGCTGTCTGCCCTGGTGATC
PagIAA9	Potri.002G108000	GGGGACAACTTTGTACAAAAAAGTTGGAATGTCTCCACCACTACTTGGTGTT	GGCGGCCGCACAACTTTGTACAAGAAAGTTGGGTATCAGAACAGTAGTAAAATTGGCTTG
PagIAA16.1	Potri.005G053900	GGGGACAACTTTGTACAAAAAAGTTGGAATGACCAGCATAATGGGAGCA	GGCGGCCGCACAACTTTGTACAAGAAAGTTGGGTATCAAAGCTCAATGAGCCTGCATGTT
PagIAA16.2	Potri.013G041400	GGGGACAACTTTGTACAAAAAAGTTGGAATGACTAGCATAATGGGAGCAGAA	GGCGGCCGCACAACTTTGTACAAGAAAGTTGGGTATCAAATTCTGTTCTTGCACTTCTCT
PagIAA16.3	Potri.005G218300	GGGGACAACTTTGTACAAAAAAGTTGGAATGGAAGTAGAGAAGGGAACAAAGA	GGCGGCCGCACAACTTTGTACAAGAAAGTTGGGTACTACTTGAAGCTTCTGTTCTTGCAT

PagIAA27.1	Potri.006G161400	GGGGACAACTTTGTACAAAAAAGTTGGAATGTCTATGCCCTTAGAGCATGAT	GGCGGCCGCACAACTTTGTACAAGAAAGTTGGGTACTAGTTACGACTTTTGCACTTCTCC
PagIAA29.2	Potri.018G127800	GGGGACAACTTTGTACAAAAAAGTTGGAATGGAACTTCAACTTGGTCTCGGTC	GGCGGCCGCACAACTTTGTACAAGAAAGTTGGGTATCAGCTGCTTCTCCTCATCAATTTT
PagIAA29.3	Potri.006G066600	GGGGACAACTTTGTACAAAAAAGTTGGAATGGAACTTCAACTTGGTCTCGGTC	GGCGGCCGCACAACTTTGTACAAGAAAGTTGGGTATCAATTGCTGCTCCTCATCAGTTTT
PagIAA12.1	Potri.010G065200	GGGGACAACTTTGTACAAAAAAGTTGGAATGATGGAAGGCTGTCTTGGTTTA	GGCGGCCGCACAACTTTGTACAAGAAAGTTGGGTACTAAACAGGCTTGCTTCTTTGTTTC

Table S4. The stability of reference genes evaluated by different algorithms.

Reference gene	GeNorm (M value)	NormFinder	BestKeeper	
			std dev [+/- CP]	CV [% CP]
PagUBQ	0.634 (1)	0.009 (1)	0.34 (2)	1.86
PagACTIN	0.649 (2)	0.012 (3)	0.34 (1)	1.62
PagPP2A-2	0.662 (3)	0.011 (2)	0.57 (6)	2.09
PagHistone	0.667 (4)	0.013 (4)	0.49 (4)	1.93
PagGAPDH	0.675 (5)	0.014 (6)	0.35 (3)	1.5
PagRPS18	0.743 (6)	0.013 (5)	0.68 (7)	2.18
PagEF1A	0.784 (7)	0.015 (7)	0.78 (8)	2.37
Pagβ-tubulin	0.876 (8)	0.025 (8)	0.54 (5)	1.93
PagCYP 59	1.016 (9)	0.028 (9)	1.04 (9)	3.25
PagEF1B	1.115 (10)	0.033 (10)	1.11 (11)	3.54
PagPP2A-A2	1.180 (11)	0.037 (11)	1.08 (10)	3.79

1-11：The rankings of the reference genes.

Table S5. The primer sequences used in real-time quantitative PCR.

Genes	Gene ID	Primer sequence F (5' to 3')	Primer sequence R (5' to 3')
Pag26S-4	Potri.015G007800	GTGCAGTGGAGCAATGAGAA	CCATGGTAGCTGGATGTGTG
Pag26S-8	Potri.007G020800	CACTCCGCTTTCTCTGTTCC	TGGGGTATTAAATGGCCAAA
PagGH3.1	Potri.007G050300	GCCCCAACGAGACAATTCTA	TTGGCAAGCTCTTTCCAGTT
PagGH3.6	Potri.014G136800	GTTCCACCACGACCTGAAGT	GCACAGAGCAGATGGCAATA
PagARF5.2	Potri.005G236700	GTGGTTCTGTGGTCGATCCT	AGGAAAGTCTTGCCGTGAGA
PagARF5.1	Potri.002G024700	CAATGCAACCACCATCTCAG	CTCACACCCACCATCAAGTG
PagP450-705A5	Potri.001G270900	GGAGCAGCCAGATGAAGAAG	ATTATCTTCCCCCGAACACC
PagE3-HOS1	Potri.001G155200	CAGCTGCAATTAAGCGTTCA	CTTGCAAAACAACCCTCCAT
PagE3-PUB24	Potri.016G069500	TGTTGTTGTGCTGAGGGAAG	GGATCACCATGCTTGTTCCT
PagUBQ	BU879229	GTTGATTTTTGCTGGGAAGC	GATCTTGGCCTTCACGTTGT
PagFBL1	KY020444	GCCTCTTCTTTCAAGGATCG	AAGCAGCATCCAATAGCTCA
PagIAA16.4	Potri.002G044900	CCTCCGTAAAGTGGACCTGA	CCAATCACCGTCCTTGTCTT
PagIAA28.1	Potri.018G057000	AACTCTCTGTTGCCATAGATGAACT	GTTGTTACCAACTGAAAGTTGTGGA
PagIAA28.2	Potri.006G236200	CCCAGTTGGAGCAAAAACAT	TGGCTTGGGCTTCTGTAACT
PagFBL1	KY020444	CACCATGTGTTGTCTGGGTG	TTCTGACCCAGCAGCTTACA
PagIAA7.1	Potri.010G078300	AAGAACCACCTTCCTGCTGA	ACTGGCGTTCTTCTGAGTCA
PagIAA7.2	Potri.008G161200	AGAACCACCTTCCTGCTGTT	ACCATCCATGCAGACCTTCA
PagIAA19.1	Potri.001G177500	AACGAGTCAAGTTGTGGGGT	CGCGACAACAAGATCCGAAT
PagIAA20.1	Potri.002G186400	TGAGTGCTGTAGTTCCACCT	GGCATTTCCCGGAATGTTCA
PagIAA9	Potri.002G108000	AAGGCAGGACAAGAGAGACC	GGCCCTGCTTTACCATCAAC
PagIAA16.1	Potri.005G053900	GAGGCTGCAAAGGGTAATGG	GTTGCTGTTCTTCTGGACGG
PagIAA16.2	Potri.013G041400	GGCCTCTACTGATCCTGCAA	GCCTAGAGCGTCAGAGAGTT
PagIAA16.3	Potri.005G218300	ATGCGGTTTGAGGAGACAGA	TAGCTGGAGGTTTTGCTGGA
PagIAA27.1	Potri.006G161400	TGGCAGCTACATGGAACTCT	CGGTGAACATCTTCCAAGGC
PagIAA29.2	Potri.018G127800	GATGGGCCTCAAACGTTACC	CGATTGATCTCTAGCGCCCT
PagIAA29.3	Potri.006G066600	GGTCTCGGTCTTCCAAGTGA	CATCTTCCTCGTTTGGCTGG
PagIAA12.1	Potri.010G065200	CATCTTGGATTTGTCAAGGTGA	ACGACCCATCCAATAGCTTAGA
PagACTIN	Potri.001G309500	AAACTGTAATGGTCCTCCCTCCG	GCATCATCACAATCACTCTCCGA
PagPP2A-2	Potri.010G127500	ATGAATTTCCTGATGTGCGACT	CAATGCCTATCCTCTGCAAGCTC
PagHistone	Potri.005G072300	ACTGTTGCTCTTCGTGAAATCCGTA	CTTAAAATCCTGGGCAATTTCACGAAC
PagGAPDH	Potri.012G094100	AACCGACTTCATTGGTGACAACCG	CCACTCATTGTCATACCACGCAAC
PagRPS18	Potri.006G170500	AGGCTCATCATCTTATCAAATCCCT	TCAATGCCACCAAATATTCGTTGCT
PagEF1A	Potri.006G275500	TACATCTTTCGGGACGGATAACCCAA	TTCAGGACTCTCATCCAATGCCCAA
Pagβ-tubulin	Potri.001G464400	CGTGCTGTTCTTATGGATCTTGAACCTG	TGCCCTTTAGCCCAGTTATTACCAG

PagCYP59	Potri.005G097500	GAGACAAAGCCTCCTGATAATGTCC	CCGATATAACAGTTCCAAAGCGTGA
PagEF1B	Potri.001G225200	ACATCTAATCATCCAGTCCGGCAAG	TCGCCTCTCCTTTTGACGCTGA

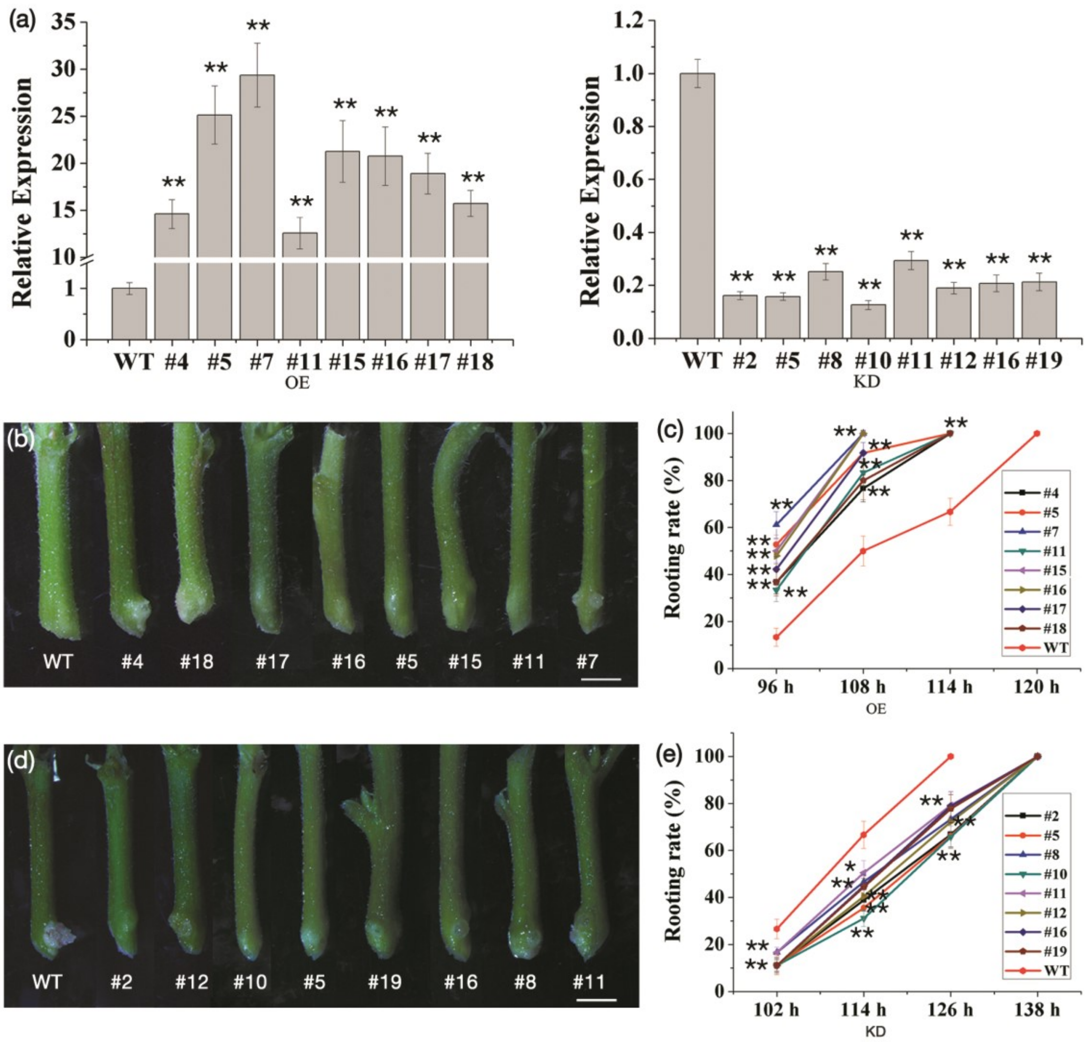

Figure S1. AR formation in 8 *PagFBL1* OE and KD lines. (a) Expression of *FBL1* by qRT-PCR analysis respectively. (b, c) AR rooting rates from leafy stems of 8 OE lines. (d, e) AR rooting rates of 8 KD lines. Bars = 1 cm. The values are means $\pm$ SE of 3 replicates. Significant differences between WT and transgenic lines are indicated with asterisks (* $P < 0.05$ and ** $P < 0.01$).

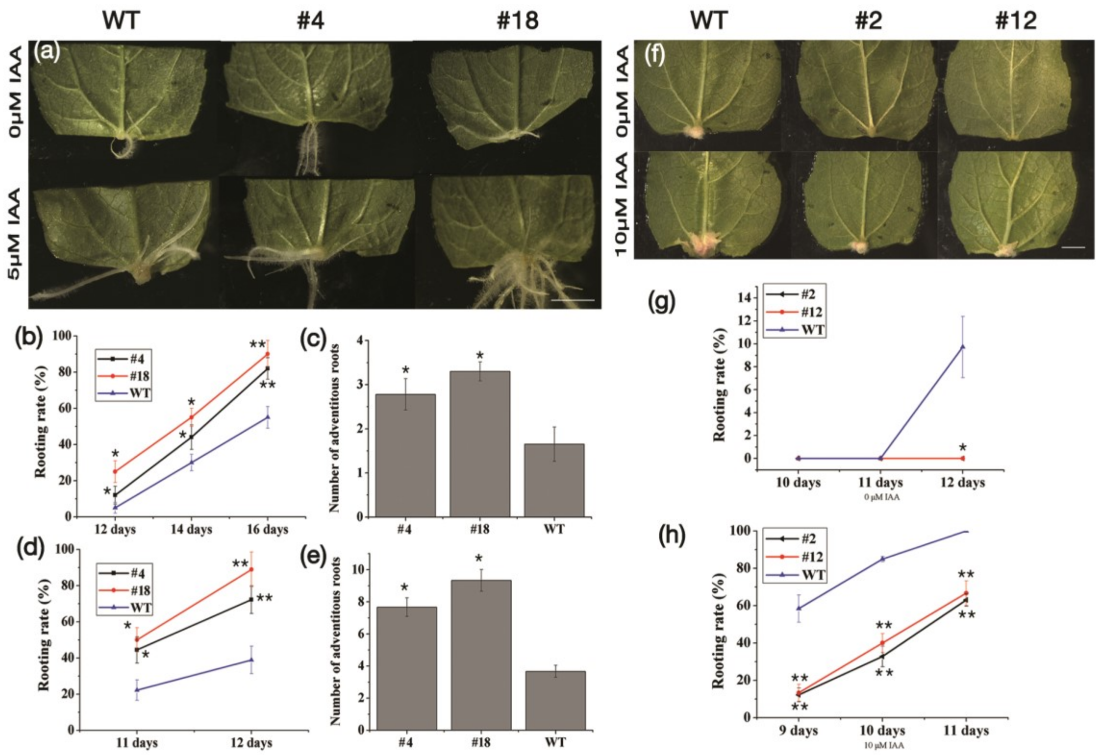

Figure S2. ARs from leaves of WT, *PagFBL1* OE lines #4 and #18 and KD lines #2 and #12 treated with (d, e, h) or without (b, c, g) IAA. (b, d) The induction rates of AR of WT and OE lines accessed during 11 and 16 days. (a, c, e) The number of ARs counted after 1 month for OE and WT lines. (f, g, h) ARs induction rate of WT and KD lines treated with or without IAA (10μM). Bars = 5 mm. The values are means ± SE of 3 replicates. Significant differences between WT and transgenic lines are indicated with asterisks (* $P < 0.05$ and ** $P < 0.01$).

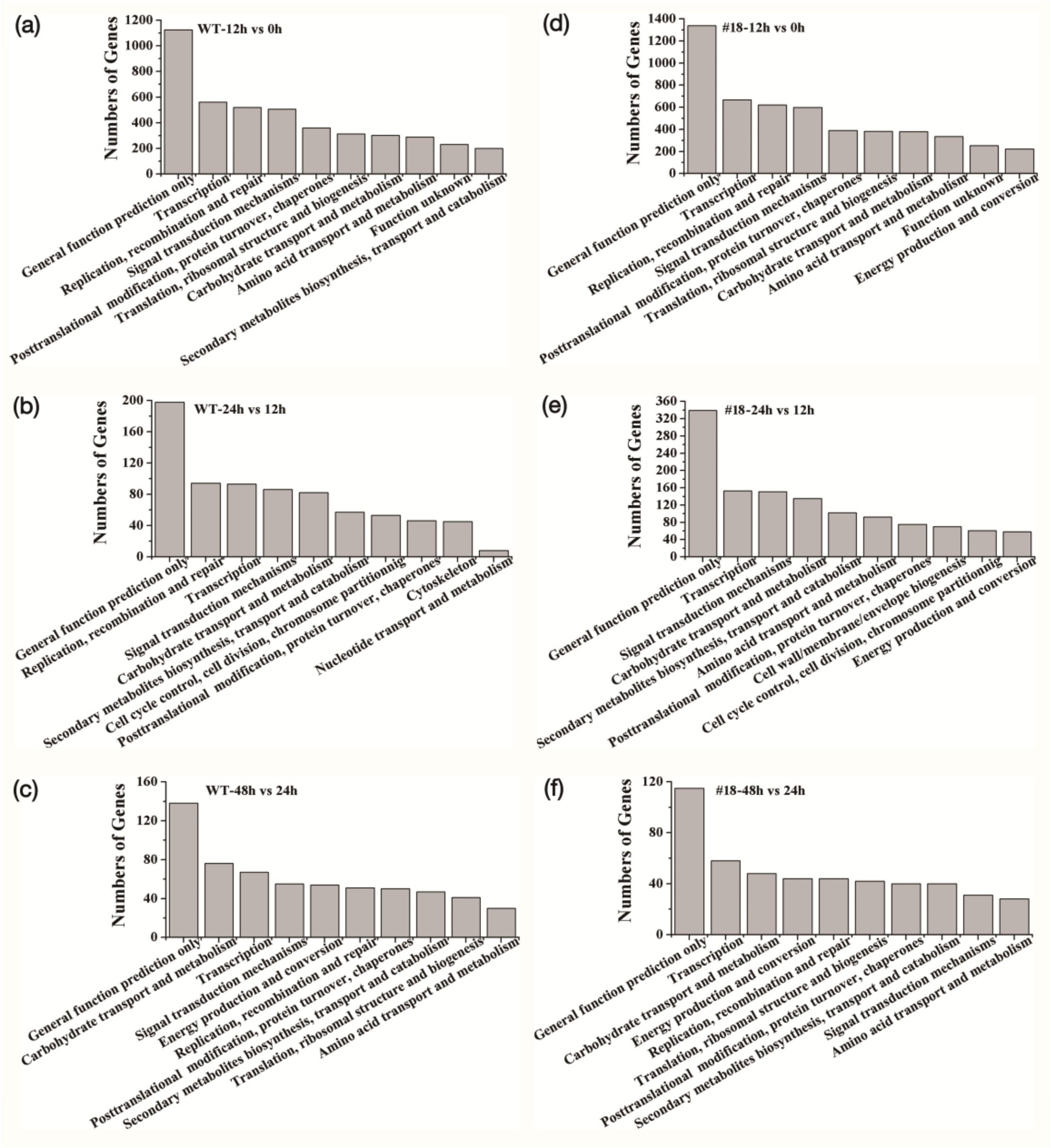

Figure S3. COG classification of DEGs. (a) 12 h vs 0 h in WT. (b) 24 h vs 12 h in WT. (c) 48 h vs 24 h in WT. (d) 12 h vs 0 h in #18. (e) 24 h vs 12 h in #18. (f) 48 h vs 24 h in #18.

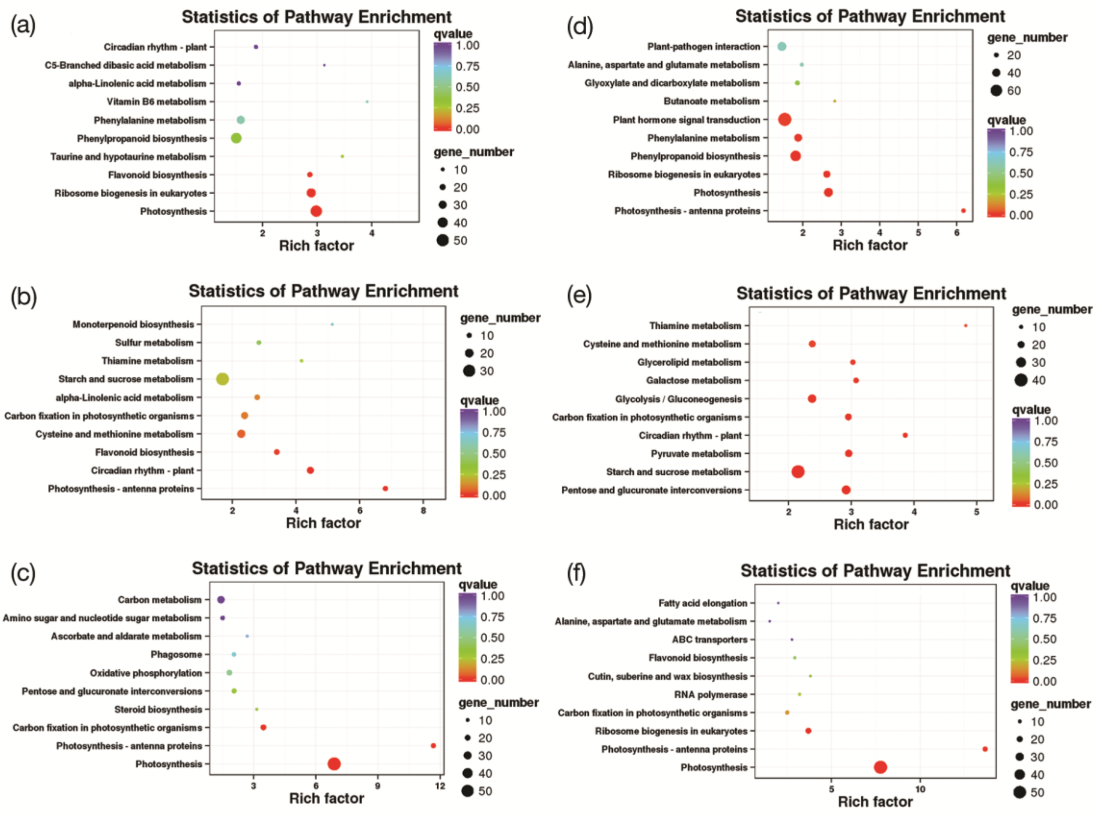

Figure S4. KEGG pathway of DEGs. (a) 12 h vs 0 h in WT. (b) 24 h vs 12 h in WT. (c) 48 h vs 24 h in WT. (d) 12 h vs 0 h in #18. (e) 24 h vs 12 h in #18. (f) 48 h vs 24 h in #18.

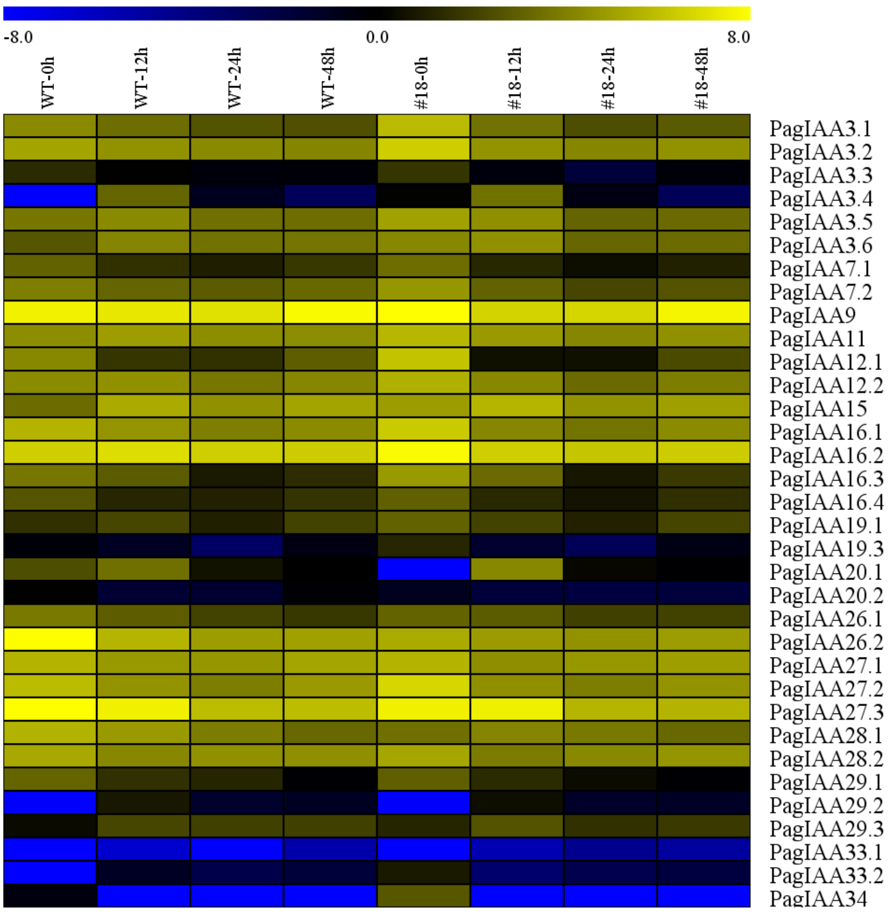

Figure S5. Heat map showing the expression patterns of *PagIAAs* during AR formation from OE transgenic line (#18) and WT at four time points.

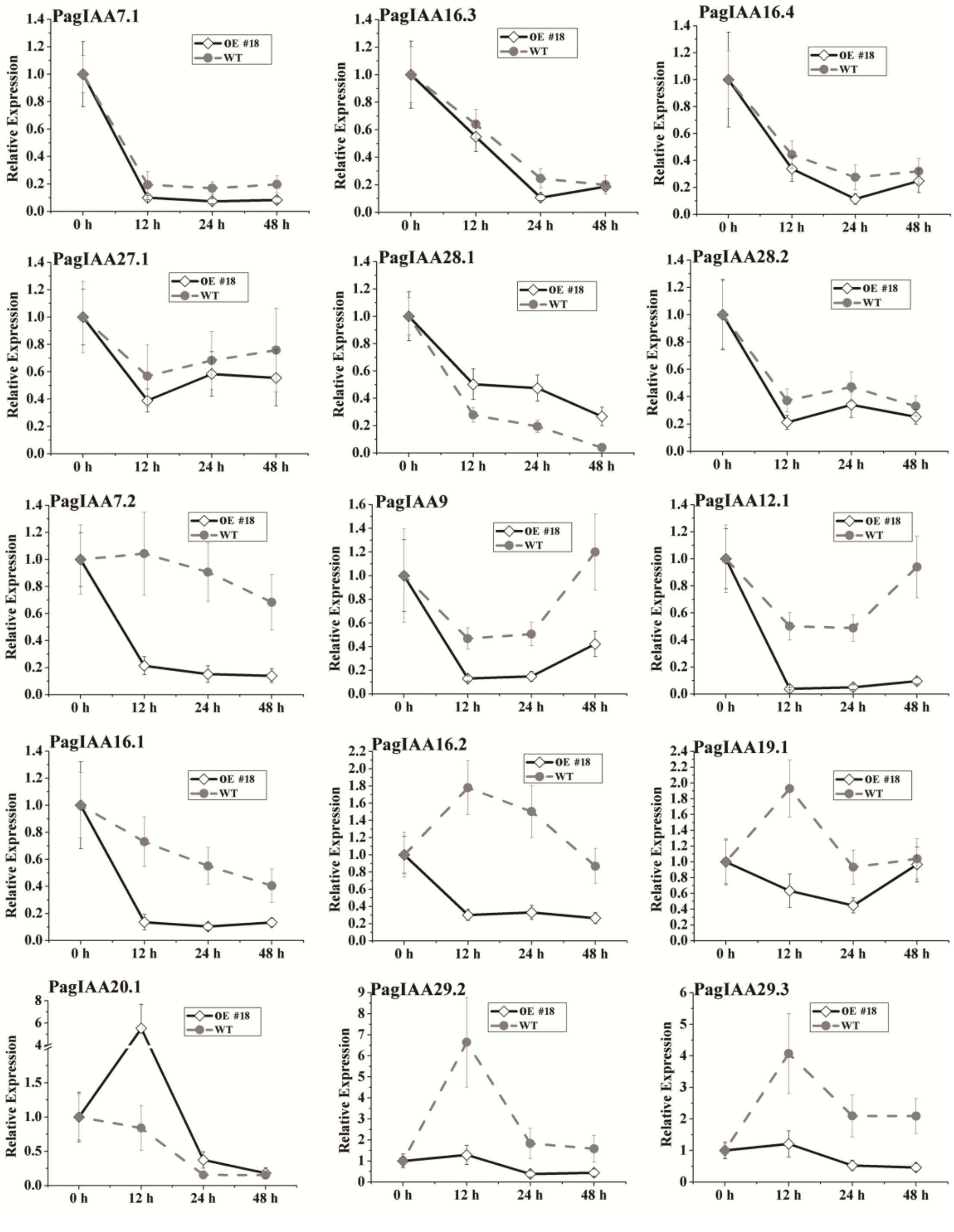

Figure S6. The expressions of 15 *PagIAAs* during AR induction by qRT-PCR.

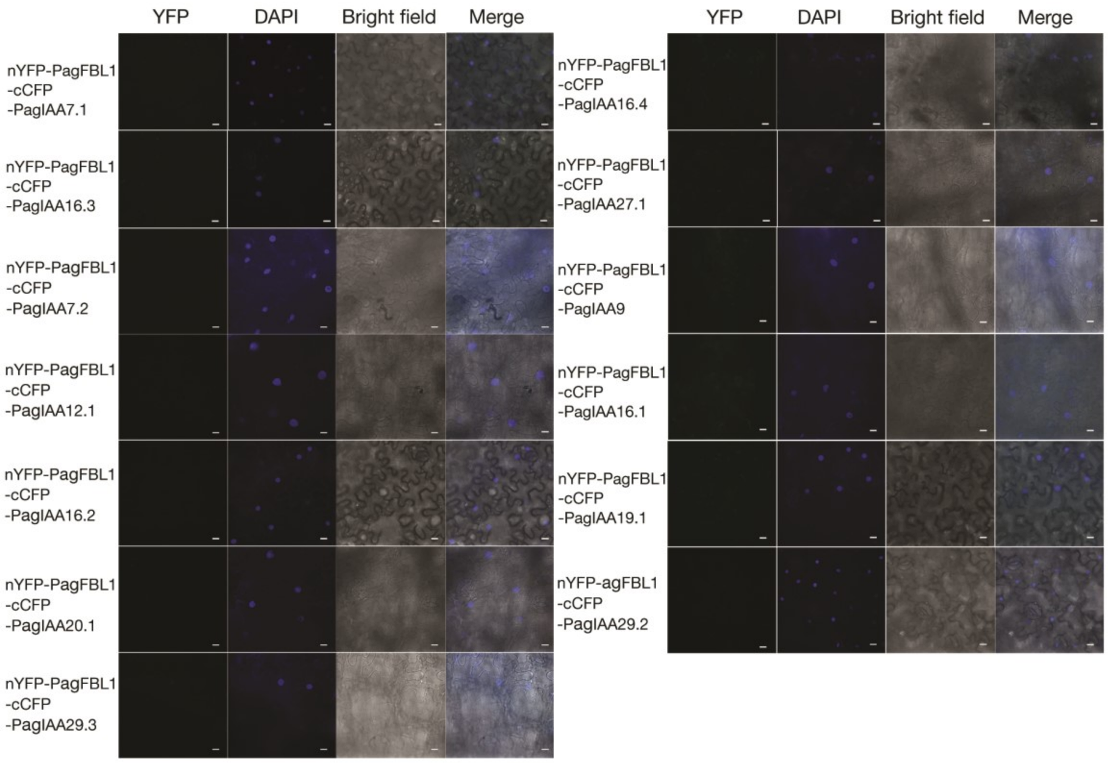

Figure S7. Interaction between PagFBL1 and PagIAA7.1, 7.2, 9, 12.1, 16.1, 16.2, 16.3, 16.4, 19.1, 20.1, 27.1, 29.2 or 29.3 with 100 μM NAA, respectively. Bars = 12 μm.
